# PARP Activity Is Essential for Retinal Photoreceptor Survival in the Human Homologous *Rho* ^I255del^ Mouse Model for Autosomal Dominant Retinitis Pigmentosa

**DOI:** 10.1111/jnc.70319

**Published:** 2025-12-22

**Authors:** Yu Zhu, Azdah Hamed A. Fallatah, Kangwei Jiao, Mathias W. Seeliger, François Paquet‐Durand

**Affiliations:** ^1^ Cell Death Mechanisms Group, Institute for Ophthalmic Research University of Tübingen Tübingen Germany; ^2^ Graduate School for Cellular and Molecular Neuroscience University of Tübingen Tübingen Germany; ^3^ Key Laboratory of Yunnan Province, Yunnan Eye Institute, Affiliated Hospital of Yunnan University Yunnan University Kunming Yunnan China; ^4^ Division of Ocular Neurodegeneration, Institute for Ophthalmic Research University of Tübingen Tübingen Germany

**Keywords:** apoptosis, cGMP, IRD, non‐apoptotic cell death, PARthanatos, RD

## Abstract

Retinitis Pigmentosa (RP) is a group of rare, inherited, neurodegenerative diseases of the retina that primarily affect rod photoreceptors. The initial loss of rods is followed by a secondary cone photoreceptor degeneration and eventually legal blindness. Despite several attempts, RP still remains essentially untreatable. In recent years, inhibition of poly (ADP‐ribose) polymerase (PARP) has been proposed as a potential therapeutic strategy for autosomal‐recessive RP, based on promising work in preclinical animal models. However, the effects of PARP inhibitors in autosomal‐dominant RP are still largely unknown. Here, we employed a novel, human‐homologous rhodopsin‐mutant *Rho* ^I255del/+^ mouse model for autosomal dominant RP to assess the impact of different PARP inhibitors on the progression of photoreceptor degeneration. The PARP inhibitors used –olaparib, saruparib, INO1001, and nicotinamide–target different PARP isoforms, and their potentially differential effects were evaluated in organotypic retinal explants cultivated under entirely defined conditions. Readouts comprised in situ activity assays for PARP and calpain‐type proteases, the TUNEL assay for cell death, as well as immunostaining for activated calpain‐2, activated caspase‐3, rhodopsin, and cone arrestin‐3. Unexpectedly, and in contrast to previous findings in animal models for recessive RP, all of the PARP inhibitors used led to marked and dose‐dependent rod photoreceptor toxicity in the *Rho* ^I255del^ dominant RP model. Furthermore, this effect appeared to be independent of rhodopsin expression. On the other hand, cone photoreceptors were apparently unaffected by PARP inhibition. The present study thus demonstrates the importance of PARP activity for rod photoreceptor viability in a dominant rhodopsin mutant, highlights the need for a deeper understanding of the mechanisms underlying photoreceptor degeneration in different RP forms, and cautions against the indiscriminate use of PARP inhibitors for the treatment of RP.

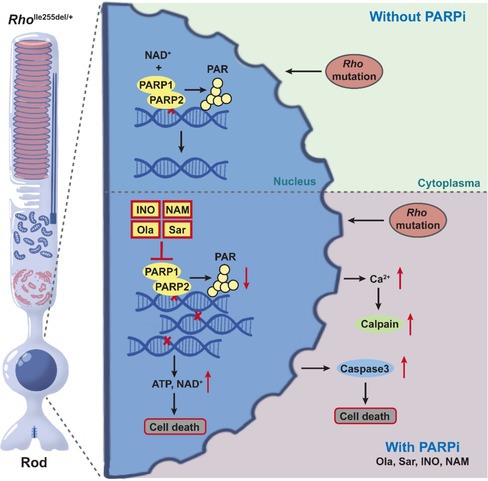

## Graphical abstract

Inhibition of PARP with different selective inhibitors (PARPi). Olaparib (Ola), Saruparib (Sar), INO1001 (INO), and nicotinamide (NAM) all increase photoreceptor cell death in the *Rho* ^I255del/+^ mouse model for autosomal dominant retinitis pigmentosa.

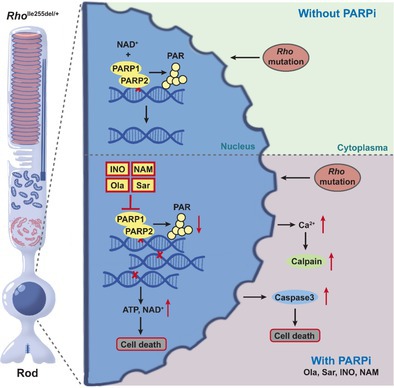

**Abbreviations**
Arr‐3: arrestin‐3
BM: basal R16 retinal culture medium
BSA: bovine serum albumin
CM: complete R16 medium
CRB: calpain reaction buffer
DMSO: dimethyl sulfoxide
ER: endoplasmic reticulum
FBS: fetal bovine serum
INL: inner nuclear layer
INO: INO1001
IRD: inherited retinal degeneration
IS: inner segments
NAD^+^
: nicotinamide adenine dinucleotide
NAM: nicotinamide
NGS: normal goat serum
NT: non‐treated
Ola: olaparib
ONL: outer nuclear layer
OS: outer segments
P: post‐natal
PAR: poly (ADP‐ribose)
PARP: poly (ADP‐ribose) polymerase
PBS: phosphate buffered saline
PBST: phosphate buffered saline + Triton X‐100
PFA: paraformaldehyde
*RHO*
: rhodopsin
RP: retinitis pigmentosa
RPE: retinal pigment epithelium
RT: room temperature
Sar: saruparib
SD: standard deviation
SPF: specified pathogen free
TBS: Tris‐buffered saline
TUNEL: terminal deoxynucleotidyl transferase dUTP nick end labeling
UPR: unfolded protein response
WT: wild‐type

## 1 Introduction

Retinitis pigmentosa (RP) refers to a group of inherited retinal degenerations (IRD), in which mutations in a large variety of genes cause primary rod photoreceptor death. Since rods are responsible for dim light vision, this initially causes night blindness. Subsequently, cone photoreceptors also succumb to degeneration, eventually resulting in complete blindness (Kaplan et al. 2017). While about 50%–60% of RP cases show autosomal recessive inheritance, 30%–40% of patients suffer from autosomal dominant disease (Bunker et al. 1984; Grøndahl 1987; Novak‐Lauš et al. 2002). Within the autosomal dominant group, approx. 25% of cases are caused by mutations in the rhodopsin (*RHO*) gene (Hartong et al. 2006). To this day, no effective treatments exist for most forms of RP.

A great number animal models are available to study RP treatment development (Arango‐Gonzalez et al. 2014; Power et al. 2020). Across many of these disease models, both for recessive and dominant disease, an over‐activation of poly (ADP‐ribose) polymerase (PARP) has been found (Arango‐Gonzalez et al. 2014; Jiao et al. 2016; Kaur et al. 2011). The 17‐member PARP family of enzymes plays an important role in promoting DNA repair and regulating gene transcription. Notably, the synthesis of poly (ADP‐ribose) (PAR) polymers is seen as a crucial factor that helps unwind chromatin structure and facilitate DNA access of repair enzymes and transcriptional machinery (Bai 2015). Paradoxically, excessive activation of PARP is linked to cell death (Park et al. 2022), including in retinal photoreceptors (Arango‐Gonzalez et al. 2014), a phenomenon that may be related to an excessive consumption of nicotinamide adenine dinucleotide (NAD^+^). Accordingly, PARP inhibitors, such as olaparib (brand name Lynparza) or INO1001, have previously been reported to provide photoreceptor neuroprotection in different models for recessive RP in vitro (Dong et al. 2023; Sahaboglu et al. 2016; Yan et al. 2022) and in vivo (Sahaboglu et al. 2016). However, the efficacy of PARP inhibitors in protecting photoreceptors in dominant forms of RP is still largely unclear.

We employed the recently generated human homologous *Rho* ^I255del^ knock‐in mouse model for dominant RP (Cao, Dahlen, et al. 2024; Cao, Zhu, et al. 2024; Zhu, Kumar, et al. 2025; Zhu, Peiroten, et al. 2025) to test different PARP inhibitors for potential neuroprotective effects. An earlier study found high PARP activity in *Rho* ^I255del/+^ photoreceptors. This activity peaked at post‐natal (P) day 18, preceding the peak of cell death by 2 days and suggesting PARP activation as an early event in the degenerative process (Cao, Dahlen, et al. 2024; Cao, Zhu, et al. 2024). In the present study, we found PARP inhibitors to exert strong negative effects on *Rho* ^I255del^ rod photoreceptors. These results point to possible differences in degenerative mechanisms between recessive and dominant RP and suggest that PARP inhibition as a potential therapeutic strategy may need to be tested and tailored to individual disease genotypes.

## 2 Materials and Methods

### 2.1 Animals

*Rho* ^l255del/+^ (*Rho* ^l255d/+^) mice carry a human homologous gene defect that results in the deletion of one of the two isoleucine residues located in positions 255 and 256 of the rhodopsin protein (Cao, Dahlen, et al. 2024; Cao, Zhu, et al. 2024). The study employed heterozygous C57BL/6J *Rho* ^l255del/+^ mice that were generated by crossing homozygous *Rho* ^l255del/I255del^ (GenOway; Lyon, France) with C57BL/6J *Rho* ^+/+^ (RRID: MGI: 3 028 467) wild‐type (WT) animals, which were purchased from Jackson Laboratories (Bar Harbor, ME, USA). Animals were housed in a specified pathogen‐free (SPF) facility, under standard white cyclic lighting, had free access to food and water, and were used irrespective of sex. WT and mutant mouse lines are regularly intercrossed to ensure congenicity. All efforts were made to minimize the number of animals used. To limit the pain and suffering of animals, and to reduce the overall numbers of animals required, all experiments were conducted using in vitro organotypic retinal explant cultures. All procedures were performed in accordance with the ARVO statement for the use of animals in ophthalmic and visual research. Animal protocols compliant with §4 of the German law of animal protection were reviewed and approved by the Tübingen University committee on animal protection (Einrichtung für Tierschutz, Tierärztlicher Dienst und Labortierkunde, Registration No. AK02/19M). A total of 88 animals (166 retinal explants) were used for the study.

### 2.2 Organotypic Retinal Explant Cultures

Retinal explantation and cultivation under sterile conditions followed previously published protocols (Belhadj et al. 2020). In brief, *Rho* ^l255del/+^ and WT animals were sacrificed at P12 by use of CO_2_ asphyxiation followed by cervical dislocation. The eyes were enucleated and incubated in basal R16 retinal culture medium (BM; cat. no. 07491252A, Gibco, Paisley, UK) for 5 min at room temperature (RT). To facilitate explantation of the neuroretina with attached retinal pigment epithelium (RPE), the sclera was predigested for 17 min with 0.12% proteinase K (cat. no. P6556; Sigma–Aldrich, Hamburg, Germany) in R16 BM. To inactivate proteinase K, the eyes were incubated in R16 BM containing 20% fetal bovine serum (FBS; cat. no. F7524, Sigma) for 5 min at RT. Under a stereoscope, the eyes were then dissected in fresh R16 BM, the anterior segments, sclera, and choroid were removed, and the optic nerve was cut, leaving only the neuroretina with its RPE attached. To flatten the retina, four incisions were made, resulting in a four‐leaf clover shape. The retinal explant was then transferred to a 6‐well culture plate with polycarbonate membrane inserts (cat. no. 3412, Corning Life Sciences, NY, USA), with the RPE facing the membrane. Retinal explant cultures were maintained in 1 mL of complete R16 medium (CM) with supplements, free of serum and antibiotics (Belhadj et al. 2020; Zhu et al. 2022), in a sterile, humidified incubator at 37°C with 5% CO_2_.

**FIGURE 1:**
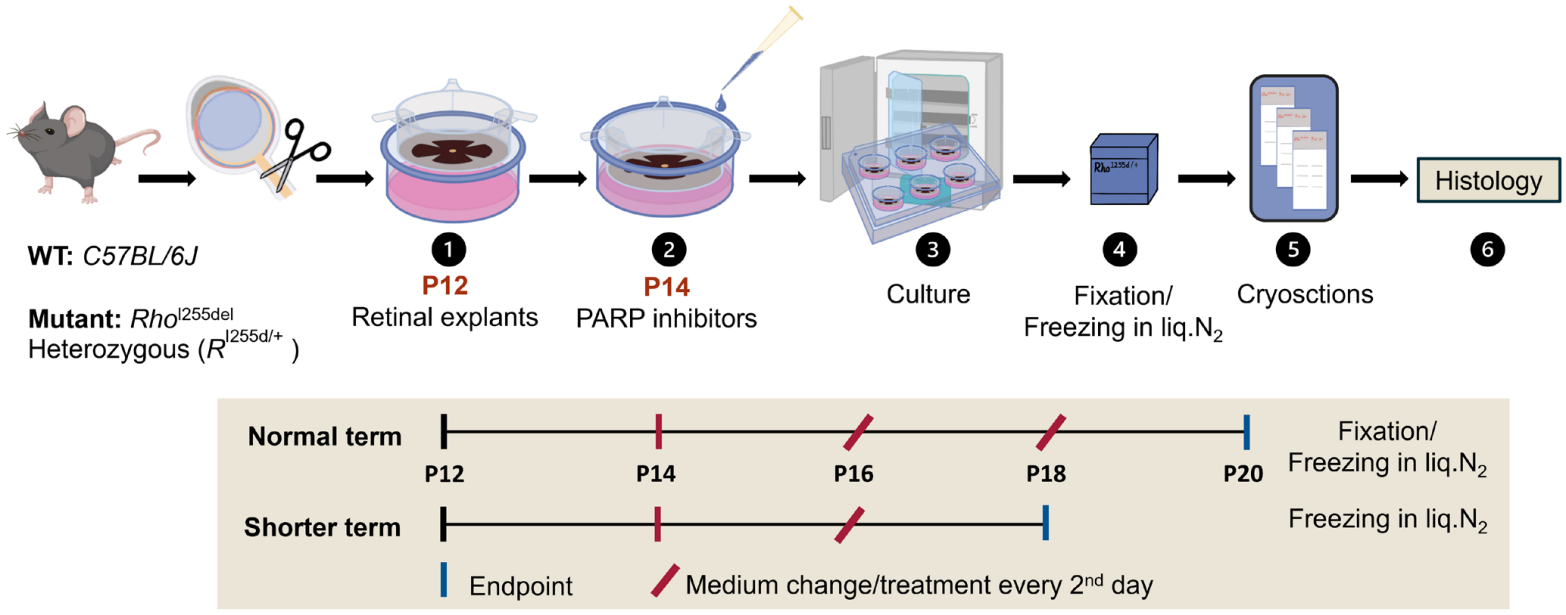
Schematic overview of experimental paradigms. Organotypic retinal explant cultures were derived from either wild‐type (WT) or *Rho* ^I255d/+^ animals at post‐natal (P) day 12 and cultured until P18 or P20. The culture medium was changed every second day, and treatment with PARP inhibitors olaparib, saruparib, INO1001, and nicotinamide (or dimethyl sulfoxide (DMSO) control) was applied from P14 onwards. Cultures ended at P18 or P20, either by 4% paraformaldehyde (PFA) fixation (fixed tissue) or by immediate freezing in liquid N_2_ (unfixed tissue).

After 48 h (i.e., at an age equivalent to P14), retinal explants were separated into non‐treated (NT) control, exposed only to CM and dimethyl sulfoxide (DMSO; cat. no. 5.89569, Sigma–Aldrich; 0.01%, 0.1%, and 1%), and treatment groups exposed to different PARP inhibitors dissolved in DMSO and CM. The PARP inhibitors olaparib (Ola), saruparib (Sar), and INO1001 (INO) were used at 0.1, 1, and 10 μM concentrations, while nicotinamide (NAM) was used at 20, 200, 1000, and 2000 μM. For chemical structures and specificities of the PARP inhibitors used see Table 1. The culture medium was changed every second day, and culture ended at either P18 or P20 (Figure 1).

**TABLE 1:** Overview of different PARP inhibitors used in this project. Information on different PARP inhibitors, including chemical structures, targets, IC_50_‐values, providers, and corresponding references.

Compounds	Chemical structure	Targets	IC_50_	Supplier (catalog no.)	References
Olaparib (Ola; AZD2281)	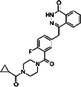	PARP‐1 PARP‐2 PARP‐3	PARP‐1: 5 nM PARP‐2: 1 nM PARP‐3: 200 nM	TargetMol, Massachusetts, USA (cat. no. T3015)	(Ngoi et al. 2021; Pires et al. 2025; Yan et al. 2022)
Saruparib (Sar; AZ5305)	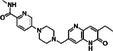	PARP‐1	PARP‐1: 3 nM PARP‐2: 1300 nM PARP‐3: 3400 nM	MedChemExpress, New Jersey, USA (cat. no. HY‐132167)	(Illuzzi et al. 2022; Johannes et al. 2021; Ngoi et al. 2021; Pires et al. 2025)
INO1001 (INO)	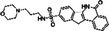	PARP‐1 PARP‐2	PARP‐1: ≈1 nM (EC_50_ = 7.9 nM) PARP‐2: ≈30 nM (EC_50_ = 217 nM)	Adooq Bioscience, California, USA (cat. no. A20680)	(Mason et al. 2008; Pires et al. 2025)
Nicotinamide (NAM, N)	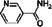	PARPs	PARP‐1: 120 μM	Merck, Darmstadt, Germany (cat. no. 72345)	(Hu et al. 2014; Li et al. 2021; Slominska et al. 2006; Zhu, Kumar, et al. 2025; Zhu, Peiroten, et al. 2025)
		HDAC class III (sirtuins)	SIRT‐1: 120 μM SIRT‐2: 100 μM SIRT‐3: 50 μM SIRT‐4: 200 μM SIRT‐5: 150 μM SIRT‐6: 184 μM		

### 2.3 Histology

Retinal explants were fixed with 4% paraformaldehyde (PFA) for 45 min, washed 3× in phosphate‐buffered saline (PBS), and then cryoprotected with 10%, 20%, and 30% graded sucrose solutions at RT. Explants with culture membrane were embedded in Tissue‐Tek O.C.T. compound (cat. no. 4583, Sakura Finetek Europe, Alphen aan den Rijn, Netherlands), snap‐frozen on liquid N_2_, and cryosectioned (12 μm thickness) on a Thermo Scientific NX50 microtome (Thermo Fisher Scientific, Waltham, MA, USA). Retinal sections were thaw‐mounted onto Superfrost Plus object slides (cat. no. J1800ARLX, R. Langenbrinck, Emmendingen, Germany) and stored at −20°C for further use. To prepare unfixed tissue, retinal explants were directly embedded in Tissue‐Tek O.C.T. (Sakura) and snap‐frozen on liquid N_2_. Compared to fixed tissue, cell nuclei in unfixed preparations may look less condensed and larger, giving an overall swollen appearance. Further processing of unfixed tissue was as above for fixed tissue.

### 2.4 TUNEL Assay

The terminal deoxynucleotidyl transferase dUTP nick end labeling (TUNEL) assay (cat. no. 12156792910; red fluorescence; Roche, Mannheim, Germany) was employed on retinal sections for the visualization of dying cells (Gavrieli et al. 1992). While fixed sections were rehydrated with phosphate‐buffered saline (PBS; 0.1 M) for 10 min, unfixed sections were first fixed by incubation in 4% PFA for 10 min and then washed in PBS (2 × 5 min). Both types of preparations were incubated with proteinase K (1.5 μg/μL) diluted in preheated Tris‐buffered saline (TBS; 1 μL enzyme in 7 mL TBS) at 37°C for 5 min. This was followed by incubation with ethanol acetic acid (70:30) at −20°C for 5 min and washing with PBS 3 times for 5 min each. Subsequently, sections were incubated with blocking solution (10% normal goat serum; NGS, 1% bovine serum albumin; BSA, 1% fish gelatin in PBS, 0.3% Triton X‐100) for 1 h at RT. TUNEL reaction solution was then applied with overnight incubation at 4°C. Afterwards, the tissue was washed in PBS (3 × 5 min) and mounted using Vectashield with DAPI (cat. no. H‐1200‐10, Vector Laboratories Inc., Burlingame, CA, USA). Here, DAPI served as a nuclear counterstain.

### 2.5 PARP in situ Activity Assay

To visualize PARP activity in individual photoreceptor cells in situ (Belhadj et al. 2021), unfixed tissue sections were dried for 30 min at 37°C, rehydrated with Tris Buffer (50 mM) for 10 min, and then incubated for 3.5 h at 37°C with PARP reaction mixture containing 1 mM dithiothreitol, 50 μM 6‐Fluo‐10‐NAD^+^ (cat. no. N023; Biolog, Bremen Germany), 10 mM MgCl_2_, 100 mM TBS, and 0.2% Triton X100. The sections were mounted in Vectashield with DAPI (Vector) for microscopy.

### 2.6 Calpain In Situ Activity Assay

This assay was performed on unfixed tissue sections (Belhadj et al. 2022). After thawing, retinal tissue sections were dried for 30 min at 37°C and then rehydrated for 15 min in calpain reaction buffer (CRB; 5.96 g HEPES, 4.85 g KCl, 0.47 g MgCl_2_, and 0.22 g CaCl_2_ in 100 mL ddH_2_O; pH 7.2). Afterwards, sections were placed in CRB with 2 mM dithiothreitol and tBOC‐Leu‐Met‐CMAC (25 μM; cat. no. A6520; Thermo Fisher Scientific) for 3.5 h at 37°C. After washing with PBS (2 × 10 min), the sections were incubated with ToPro‐3 (1:1000, cat. no. T3605, Invitrogen, Eugene, Oregon, USA) as a nuclear counterstain for 25 min. Finally, sections were mounted using Vectashield without DAPI (cat. no. H‐1000‐10; Vector) for immediate visualization by microscopy.

### 2.7 Immunostaining

Retinal sections were rehydrated with PBS for 15 min, then incubated in blocking solution (10% NGS, 1% BSA, and 0.3% PBST [PBS + 0.3% Triton X‐100]) for 1 h at RT. This was followed by incubation with primary antibody: rabbit anti‐calpain‐2 (1:200; cat. no. AB39165, Abcam, Cambridge, UK), rabbit anti‐cone arrestin‐3 (1:500; cat. no. AB15282; Merck KGaA, Darmstadt, Germany), rabbit cleaved caspase‐3 (1:100; cat. no. D175; Cell Signaling, Danvers, USA), mouse anti‐rhodopsin (1:500; cat. no. MAB5316; Merck) at 4°C overnight. Afterwards, Alexa Fluor 488 anti‐rabbit (1:300, cat. no. A11034, Molecular Probes, Eugene, Oregon, USA) or 568 anti‐mouse secondary antibodies (1:500, cat. no. A11031, Molecular Probes) were applied for 1 h at RT. Then the tissue was washed with PBS 3 × 5 min, followed by mounting in Vectashield with DAPI (Vector).

### 2.8 Microscopy

Light and fluorescence microscopy were performed on a Zeiss Imager Z.2 microscope equipped with ApoTome 2, an Axiocam 506 mono camera, and HXP‐120V fluorescent lamp (Carl Zeiss Microscopy, Oberkochen, Germany) at RT. The excitation (λExc.)/emission (λEm.) characteristics of the filter sets used for the different fluorophores were as follows (in nm): DAPI (λExc. = 369 nm, λEm = 465 nm), AF488 (λExc. = 490 nm, λEm = 525 nm), and ToPro‐3 (λExc. = 642 nm, λEm = 661 nm). Representative images were captured from the retina using a 20×/0.8 objective lens and Zen 2.3 Blue Edition software. Sections of 12 μm thickness were analyzed using a 9‐optical section ApoTome2 Z‐stack.

### 2.9 Quantification and Statistical Analysis

Pictures were captured on three entire sagittal sections from at least three different animals. The average area occupied by a photoreceptor cell (i.e., cell size) was determined by counting DAPI‐ or ToPro‐3‐stained nuclei in nine different areas of the retinal outer nuclear layer (ONL) to establish an average cell size. To compare staining intensities for rhodopsin across images and specimens, we normalized using the inner nuclear layer (INL) staining intensity as a (negative) reference. The number of positive‐labeled cells in the ONL was counted manually. Arrestin‐3‐labeled cells in the ONL were counted manually, and their numbers were represented as cells per 100 μm of retinal circumference. Arrestin‐3‐labeled outer‐ and inner‐segments (OS/IS) lengths (in μm) were measured manually, using the Zeiss Zen software. There were 17 randomly chosen specimens for each section, which were quantified (i.e., 68 measurements per specimen). No blinding was performed for image quantification.

To calculate the minimum number of animals and samples required for the statistical analyses, a power calculation based on previously observed effect sizes (Sahaboglu et al. 2016; Zhu, Kumar, et al. 2025; Zhu, Peiroten, et al. 2025) was performed using the online tool StatistikGuru, Version 1.96 (www.statistikguru.de; information retrieved August 2025). Animals were not assigned to experimental groups prior to their sacrifice; both retinal explants generated from an experimental animal were included in the analysis and always assigned to NT or different treatment groups. Errors in graphs and text are given as standard deviation (SD). Values are given as mean ± SD. Statistical analysis was performed using GraphPad Prism 10.2.3 software (GraphPad Software, La Jolla, CA, USA); one‐way and two‐way ANOVA tests with Dunnett's multiple comparisons test were performed to compare more than two groups. Levels of significance were: * = *p* ≤ 0.05, ** = *p* ≤ 0.01, *** = *p* ≤ 0.001, **** = *p* ≤ 0.0001. Shapiro–Wilk testing was used to assess normal data distribution; 157 of 168 datasets passed the normality test. Full statistical reports are presented in Tables S1–S9.

## 3 Results

### 3.1 The PARP Inhibitor Olaparib Increases *Rho* ^I255del^ Photoreceptor Cell Death

**FIGURE 2:**
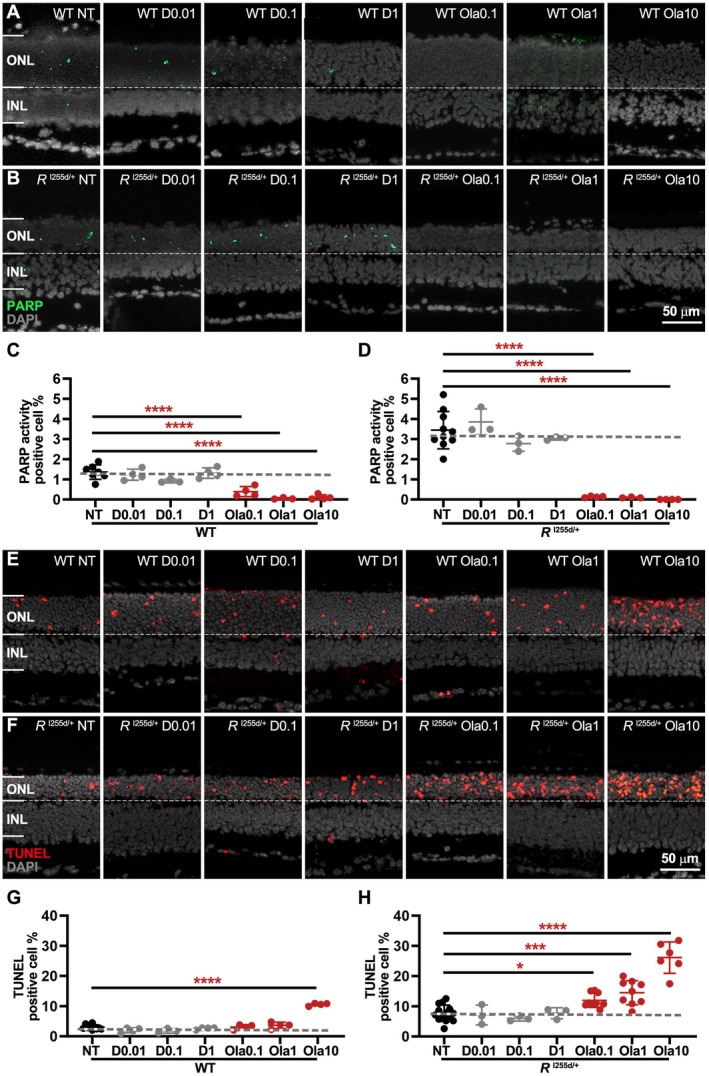
Olaparib dose‐dependently reduces PARP activity and causes photoreceptor cell death. Organotypic retinal explants were derived from wild‐type (WT) and *Rho* ^I255d/+^ (*R* ^I255d/+^) mice, and cultured from post‐natal (P) days 12 to P20. Cultures were either non‐treated (NT) or treated with different concentrations of DMSO (D; 0.01%, 0.1%, and 1%) or olaparib (Ola; 0.1, 1, and 10 μM). (A, B) PARP in situ activity assay (green) labeled cells mostly in the outer nuclear layer (ONL). (C, D) DMSO treatment did not decrease PARP activity, neither in WT nor in *Rho* ^I255d/+^ retina. In contrast, Ola significantly reduced PARP activity in both genotypes. (E, F) TUNEL assay staining dying cells (red) in the ONL of both WT and *Rho* ^I255d/+^. (G, H) Quantification of TUNEL‐positive cells showed no DMSO treatment effect in WT and *Rho* ^I255d/+^ retina, while Ola treatment dose‐dependently increased cell death. DAPI (gray) was used as nuclear counterstain. Images represent 3–10 independent retinal explant cultures; error bars indicate SD; statistical analysis: One‐way ANOVA with Dunnett's multiple comparisons test; * = *p* ≤ 0.05; *** = *p* ≤ 0.001; **** = *p* ≤ 0.0001; INL = inner nuclear layer. Scale bar = 50 μm.

Previous studies found that treatment with Ola—a PARP inhibitor that favors PARP‐2 over PARP‐1 (Table 1)—protected photoreceptors in the *rd1* mouse model for recessive RP (Dong et al. 2023; Sahaboglu et al. 2016; Yan et al. 2022), but its effect on *Rho* ^I255del^ mutant retina was unclear. To address this question, we employed organotypic retinal explants derived from heterozygous *Rho* ^I255del/+^ (*R* ^I255d/+^) and WT mice, cultured from P12 to P20. After 2 days without treatment, at P14, the explants were exposed to rising concentrations of Ola (0.1, 1, and 10 μM). Since DMSO was used as a solvent for Ola, we used DMSO‐treated cultures for comparison (0.01%, 0.1%, and 1%) (Tsai et al. 2009). Readouts consisted of PARP in situ activity on unfixed retinal sections and TUNEL assay on 4% PFA‐fixed retinal sections (Figure 2, Table S1).

In both WT and mutant retina, exposure to DMSO did not alter PARP activity in the ONL when compared to non‐treated (NT) retina. In contrast, Ola strongly reduced PARP activity in both WT and *Rho* ^I255del/+^ retinas (Figure 2A–D), indicating that it had inhibited PARP effectively.

The numbers of dying ONL cells, as detected by the TUNEL assay, were not changed by DMSO. Remarkably, the numbers of TUNEL‐positive cells in the ONL were dose‐dependently increased by Ola treatment in mutant retina and—to a lesser extent—in WT retina, when compared to NT (Figure 2E–H). At 10 μM concentration, Ola was clearly toxic to both genotypes. Since Ola targets PARP‐1 and ‐2, these results indicate that in *Rho* ^I255del/+^ retina, these PARP isoforms may be protective factors. The effect of Ola appeared to be restricted to photoreceptors, as no dose‐dependent increase in cell death in the INL was observed.

### 3.2 Olaparib Increases Caspase‐3 Activation in Rod Photoreceptors

Cell death is typically associated with increased proteolysis. In both recessive and dominant RP animal models, retinal degeneration has previously been connected to excessive activation of Ca^2+^‐dependent, calpain‐type proteases, and activation of the calpain‐2 isoform (Arango‐Gonzalez et al. 2014; Belhadj et al. 2022; Power et al. 2020). Increased calpain activity has also been found in *Rho* ^I255d/+^ retina, with peak activity at P20, that is, 2 days after the peak of PARP activity (Cao, Dahlen, et al. 2024; Cao, Zhu, et al. 2024). Calpain activity may be related to non‐apoptotic cell death, triggered by high cGMP (Power et al. 2020). In contrast, apoptotic cell death is typically connected to caspase‐type proteases, in particular to activation of caspase‐3 (Arango‐Gonzalez et al. 2014).

**FIGURE 3:**
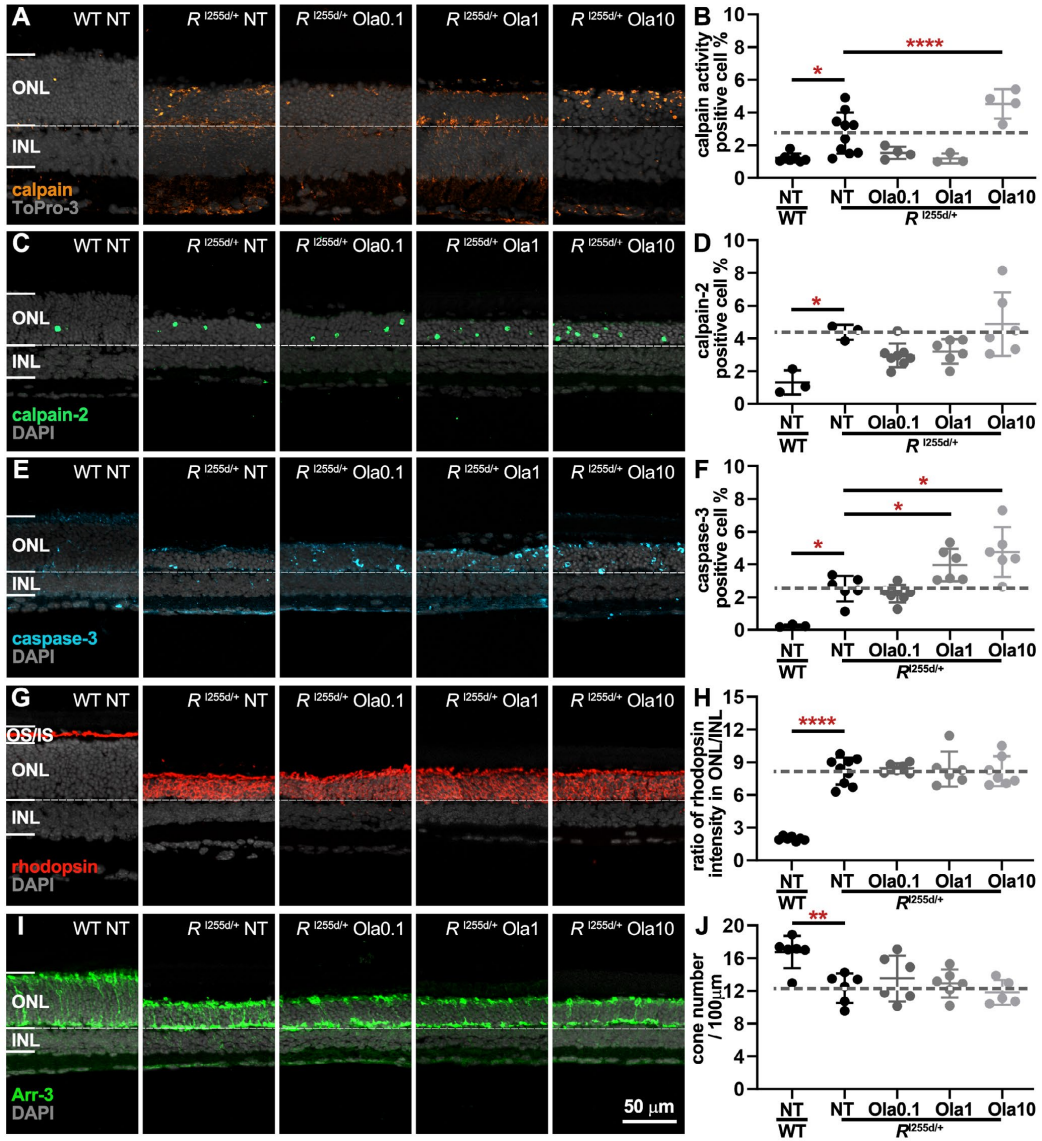
Olaparib alters proteolytic activities in *Rho* ^I255d/+^ retina, without affecting cone photoreceptors. Organotypic retinal explants were derived from wild‐type (WT) and *Rho* ^I255d/+^ (*R* ^I255d/+^) mice and cultured from post‐natal (P) days 12 to P20. Cultures were either non‐treated (NT) or treated with 0.1, 1, or 10 μM olaparib (Ola). Calpain in situ activity assay was performed on unfixed tissue sections, while immunostaining for activated calpain‐2, caspase‐3, rhodopsin, and arrestin‐3 (Arr‐3) was performed on fixed sections. (A, B) Calpain activity assay (orange) labeled cells in the outer nuclear layer (ONL), and their numbers were significantly increased at 10 μM Ola. (C, D) Compared to WT, calpain‐2 activation (green) was prominently increased in the mutant retina, however, Ola treatment did not significantly change calpain‐2. (E, F) Immunostaining for cleaved caspase‐3 (cyan) produced a ring‐shaped labeling in ONL cells, which was more prominent in the mutant compared to WT. The number of caspase‐3‐positive cells increased at 1 and 10 μM Ola compared to NT mutant retinas. (G, H) Rhodopsin immunostaining (red) revealed a mislocalization to the ONL in mutant retina, that was not seen in WT. Yet, Ola treatment did not alter the ratio of rhodopsin ONL/inner nuclear layer (INL) staining intensity. (I, J) Cone photoreceptors labeled by cone‐arrestin‐3 (Arr‐3; green) showed a decrease in NT mutant compared to WT; however, Ola treatment did not affect cones. Nuclear counterstain (gray) with ToPro‐3 (A) or DAPI (C, E, G, I). Images are representative for 3–10 independent retinal cultures; error bars indicate SD; statistical analysis: Two‐way ANOVA with Dunnett's multiple comparisons test; levels of significance were: * = *p* ≤ 0.05; ** = *p* ≤ 0.01; **** = *p* ≤ 0.0001. Scale bar = 50 μm.

To determine the effect of Ola on calpain activity, as well as on calpain‐2 and caspase‐3 activation, we subjected Ola‐treated *Rho* ^I255d/+^ retina to corresponding staining procedures (Figure 3, Table S2). In P20 cultured *Rho* ^I255d/+^ retina, the numbers of cells displaying high calpain activity in the ONL of NT mutant were significantly increased compared to WT. Yet, calpain activity was significantly increased at a concentration of 10 μM Ola, compared to NT mutant (Figure 3A,B). However, calpain‐2 activation, which was increased in NT mutant compared to NT WT, was not altered by Ola treatment in *Rho* ^I255d/+^ retina (Figure 3C,D). Cells showing activation of caspase‐3 were essentially absent from NT WT, but significantly increased in NT *Rho* ^I255d/+^ ONL. In the mutant ONL, caspase‐3 activation increased with Ola concentration (Figure 3E,F), indicating that Ola treatment may have promoted the execution of apoptotic cell death.

Rod photoreceptors in *Rho* ^I255d/+^ retina were previously found to have high levels of rhodopsin mislocalized to the ONL rather than to photoreceptor outer segments (OS) (Cao, Dahlen, et al. 2024; Cao, Zhu, et al. 2024). To assess whether Ola had altered rhodopsin trafficking, we used immunostaining and compared the rhodopsin staining intensity between INL and ONL (Figure 3G,H). In WT retina, the rhodopsin label was restricted to OS, which in the in vitro cultures appears somewhat shorter compared to the in vivo situation (Söderpalm et al. 1994). The ratio of rhodopsin staining intensity ONL/INL showed a clear increase in *Rho* ^I255d/+^ retina compared to WT. This ratio was not affected by rising Ola levels, suggesting that rod photoreceptor rhodopsin localization was not changed by the treatment.

Since in the mouse ONL rod photoreceptors outnumber cone photoreceptors approx. 30:1 (Carter‐Dawson and LaVail 1979) (i.e., cones constitute approx. 3% of all ONL cells), the very high cell death numbers seen with Ola treatment suggested that mostly rods were affected by the treatment. The previous characterization of the *Rho* ^I255d/+^ model indicated a loss of cone photoreceptors starting at P20 (Cao, Dahlen, et al. 2024; Cao, Zhu, et al. 2024). To evaluate possible effects of Ola on cone viability, we used immunostaining for the cone marker arrestin‐3 (Arr‐3). Remarkably, while the number of cones was generally decreased in mutant retina compared to WT, Ola did not alter cone survival in mutant retina. Similarly, Ola treatment did not change the length of cone OS/IS in *Rho* ^I255del/+^ retina except for 1 μM Ola (Figure S1, Table S3). Overall, these results suggest that Ola‐mediated PARP inhibition had a strong detrimental effect on rod but not cone photoreceptors. This detrimental effect appeared to be connected to increased caspase‐3‐dependent apoptosis but seemed independent of mislocalized rhodopsin.

### 3.3 Destructive Effects of Olaparib Do Not Depend on Culture Duration

The negative effect of Ola on photoreceptor degeneration at P20, while showing strong PARP inhibition, was unexpected. To investigate whether this effect was somehow influenced by the duration of the treatment, we decreased the culturing period by 2 days to end at P18, that is, the earliest time‐point at which the *Rho* ^I255del/+^ mutant displays a significant increase in PARP activity and photoreceptor cell death (Cao, Dahlen, et al. 2024; Cao, Zhu, et al. 2024). Moreover, at P18, 1 μM Ola treatment strongly reduced PARP activity in mutant retina (Figure S2A,B, Tables S4 and S5), while not affecting ONL calpain activity (Figure S2C,D). The TUNEL assay confirmed a strong increase in ONL cell death under Ola treatment (Figure S2E,F). These results indicated that PARP inhibition triggered *Rho* ^I255d/+^ photoreceptor cell death already at P18, that is, prior to the peak of cell death in the *Rho* ^I255del/+^ mutant retina. To facilitate a comparison of Ola effects in short‐ (P18) and long‐term (P20) treatment, we plotted PARP activity, calpain activity, cell death, and ONL row counts against culture duration (Figure S2G–J). There was a tendency for all four parameters to decrease from P18 to P20, reflecting the progression of the degeneration in this duration.

### 3.4 The PARP‐1 Inhibitor Saruparib Causes Photoreceptor Cell Death in *Rho* ^I255d^ ^/+^ Retina

While Ola inhibits both PARP‐1 and ‐2 isoforms, Sar is a highly selective PARP‐1 inhibitor (Table 1). To assess whether the detrimental effects observed with Ola were due to PARP‐1 or PARP‐2 inhibition, we exposed *Rho* ^I255d/+^ organotypic retinal explant cultures to Sar, using the same experimental paradigm as for Ola.

**FIGURE 4:**
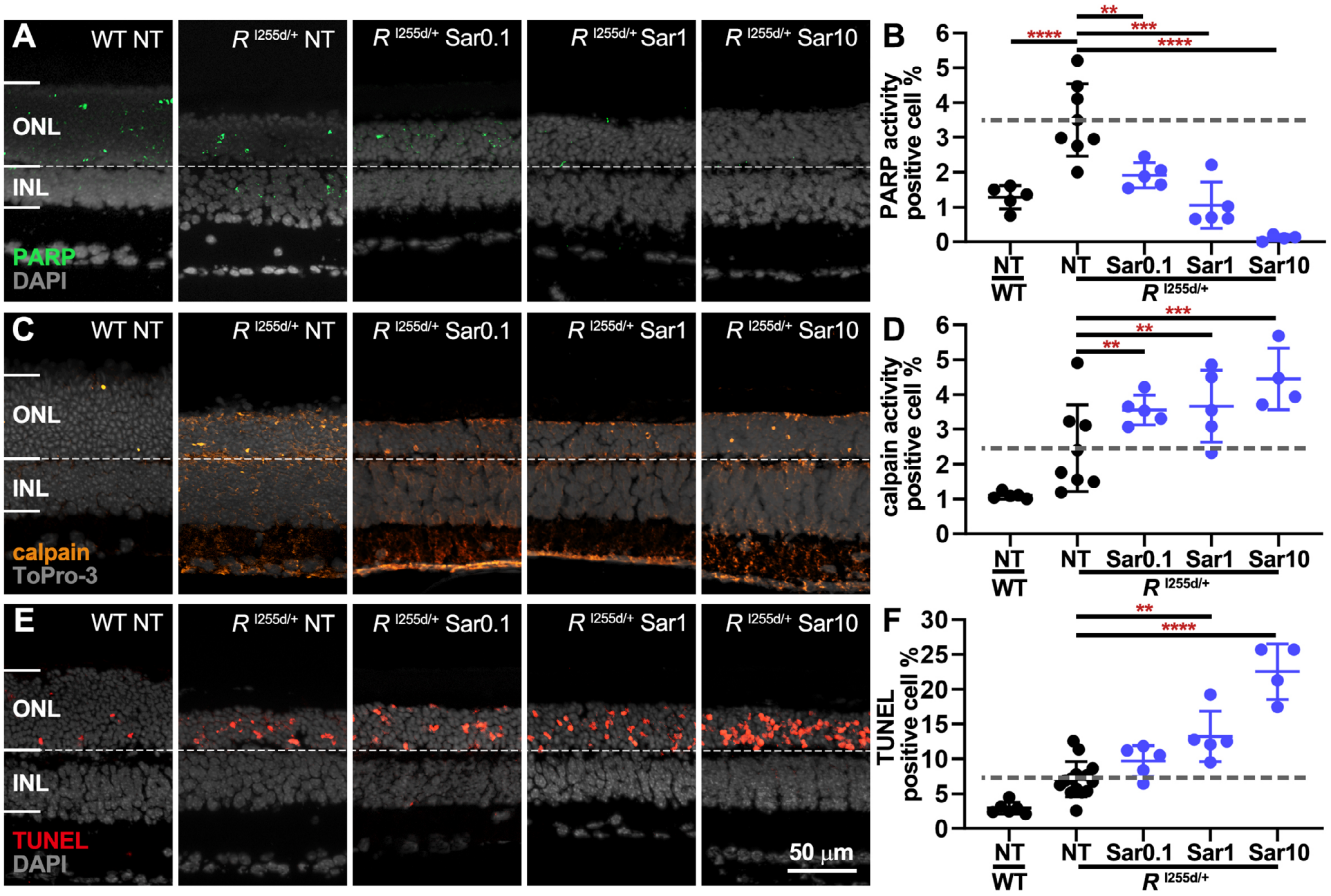
Saruparib decreases PARP activity; increases calpain activity and photoreceptor cell death. Organotypic retinal explants were derived from *Rho* ^I255del/+^ (*R* ^I255d/+^) mice and cultured from post‐natal (P) days 12 to P20. Cultures were either non‐treated (NT) or treated with 0.1, 1, or 10 μM saruparib (Sar). TUNEL‐assay, PARP‐ and calpain‐ in situ activity were used as readouts. Wild‐type (WT) data shown for comparison. (A, B) Sar treatment significantly reduced PARP‐positive cells (green) in the outer nuclear layer (ONL). (C, D) Calpain activity‐positive cells (orange) were increased by Sar treatment. (E, F) ONL cell death, visualized via TUNEL assay (red), was dose‐dependently increased by Sar treatment. DAPI (A, E) and ToPro‐3 (C) served as nuclear counterstains (gray). Images are representative for 4–14 independent retinal cultures; error bars indicate SD; statistical analysis: Two‐way ANOVA with Dunnett's multiple comparisons test; levels of significance were: ** = *p* ≤ 0.01; *** = *p* ≤ 0.001; **** = *p* ≤ 0.0001; INL = inner nuclear layer. Scale bar = 50 μm.

With Sar treatment, the number of *Rho* ^I255d/+^ ONL cells displaying high PARP activity decreased in a dose‐dependent fashion (Figure 4A,B, Table S6). Conversely, in mutant retinas, calpain activity‐positive cells increased with rising Sar concentration compared to the NT situation (Figure 4C,D). This increase in calpain activity, already at low Sar concentrations, was not seen with Ola treatment (*cf*. Figure 3B). *Rho* ^I255d/+^ ONL cell death (TUNEL assay) showed a similar Sar dose‐dependent increase (Figure 4E,F). As with Ola, no increases in INL cell death were seen. These results suggest that some of the PARP activity seen in the *Rho* ^I255d/+^ ONL likely originates from PARP‐2, and that specific PARP‐1 inhibition induces photoreceptor cell death and calpain activity in the *Rho* ^I255d/+^ retina.

### 3.5 INO1001 Inhibits PARP Activity and Triggers Photoreceptor Cell Death in *Rho* ^I255d^ ^/+^ Retina at High Doses

**FIGURE 5:**
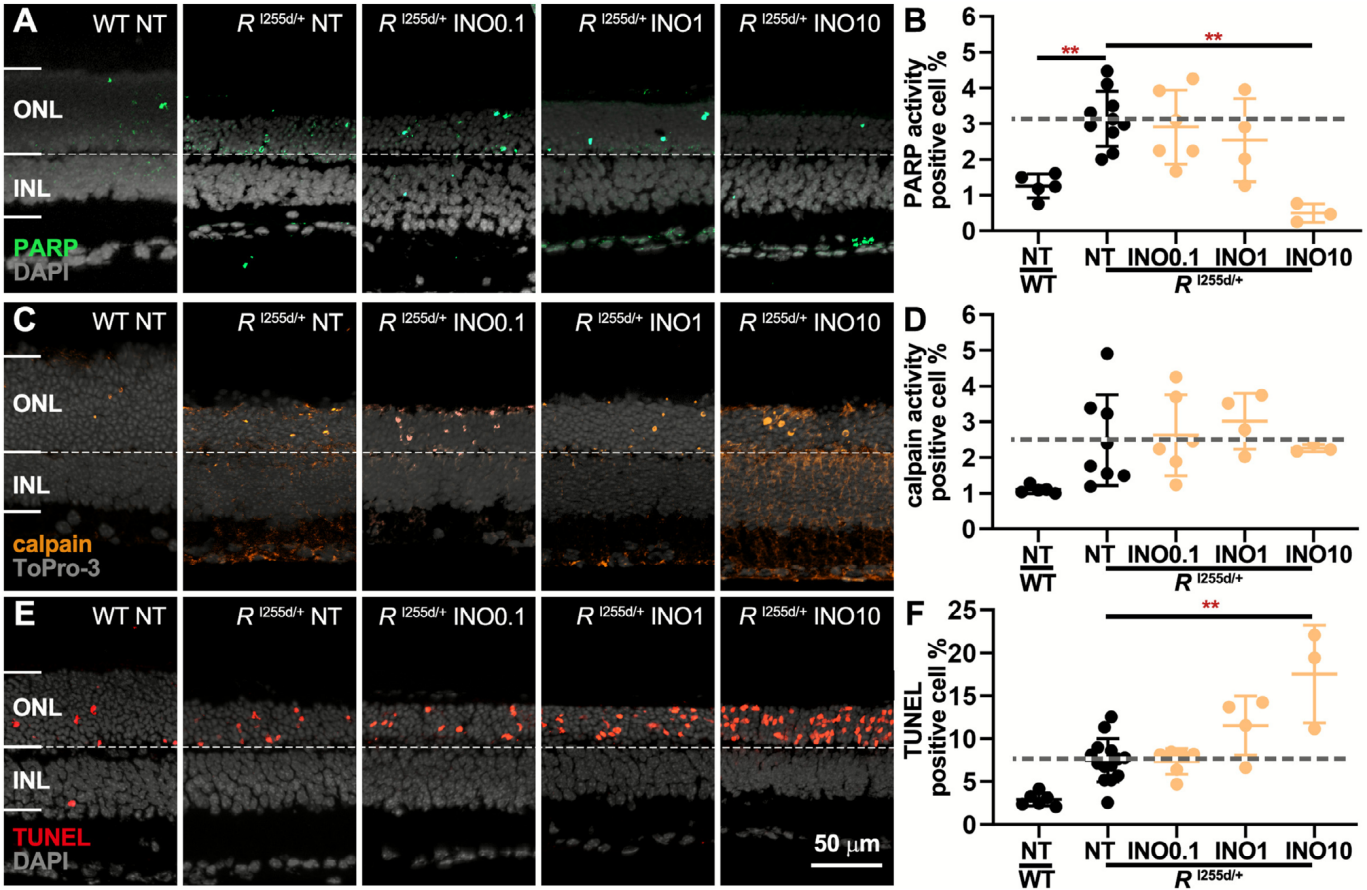
INO1001 inhibits PARP activity and induces photoreceptor cell death at highest concentration. Organotypic retinal explants were derived from *Rho* ^I255del/+^ (*R* ^I255d/+^) mice and cultured from post‐natal (P) days 12 to P20. Cultures were either non‐treated (NT) or treated with 0.1, 1, or 10 μM INO1001 (INO). PARP‐ and calpain‐ in situ activity and TUNEL assay were used as readouts. Wild‐type (WT) data shown for comparison. (A, B) The numbers of PARP‐activity positive cells (green) in the outer nuclear layer (ONL) of the mutant retina were significantly reduced by 10 μM INO treatment. (C, D) The numbers of calpain activity assay positive cells (orange) in the ONL were not changed by INO treatment. (E, F) ONL cell death (TUNEL assay, red) was significantly increased by 10 μM INO treatment. DAPI (A, E) and ToPro‐3 (C) served as nuclear counterstains (gray). Images are representative for 3–14 independent retinal cultures; error bars indicate SD; statistical analysis: Two‐way ANOVA with Dunnett's multiple comparisons test; levels of significance were: ** = *p* ≤ 0.01; INL = inner nuclear layer. Scale bar = 50 μm.

To confirm the detrimental effects of PARP inhibition, we used a third compound, INO, which displays a marked (30‐fold) preference for PARP‐1 over PARP‐2 (Pires et al. 2025) (Table 1). Using the same test paradigm as before, we found INO to have a relatively weak effect on the numbers of PARP‐activity positive cells in the *Rho* ^I255d/+^ ONL, an effect that was significant only at a 10 μM concentration (Figure 5A,B, Table S7). As opposed to the treatments with Ola and Sar, INO did not change calpain activity in the mutant ONL (Figure 5C,D). Nevertheless, INO exhibited a clear detrimental effect on *Rho* ^I255d/+^ photoreceptor viability, as indicated by the increase in the number of TUNEL‐positive cells (Figure 5E,F), something not observed in the INL. To sum up, INO inhibited PARP activity and induced photoreceptor cell death at high concentration.

### 3.6 Nicotinamide Decreases PARP Activity, Displays *Rho* ^I255d^ ^/+^ Photoreceptor Toxicity

**FIGURE 6:**
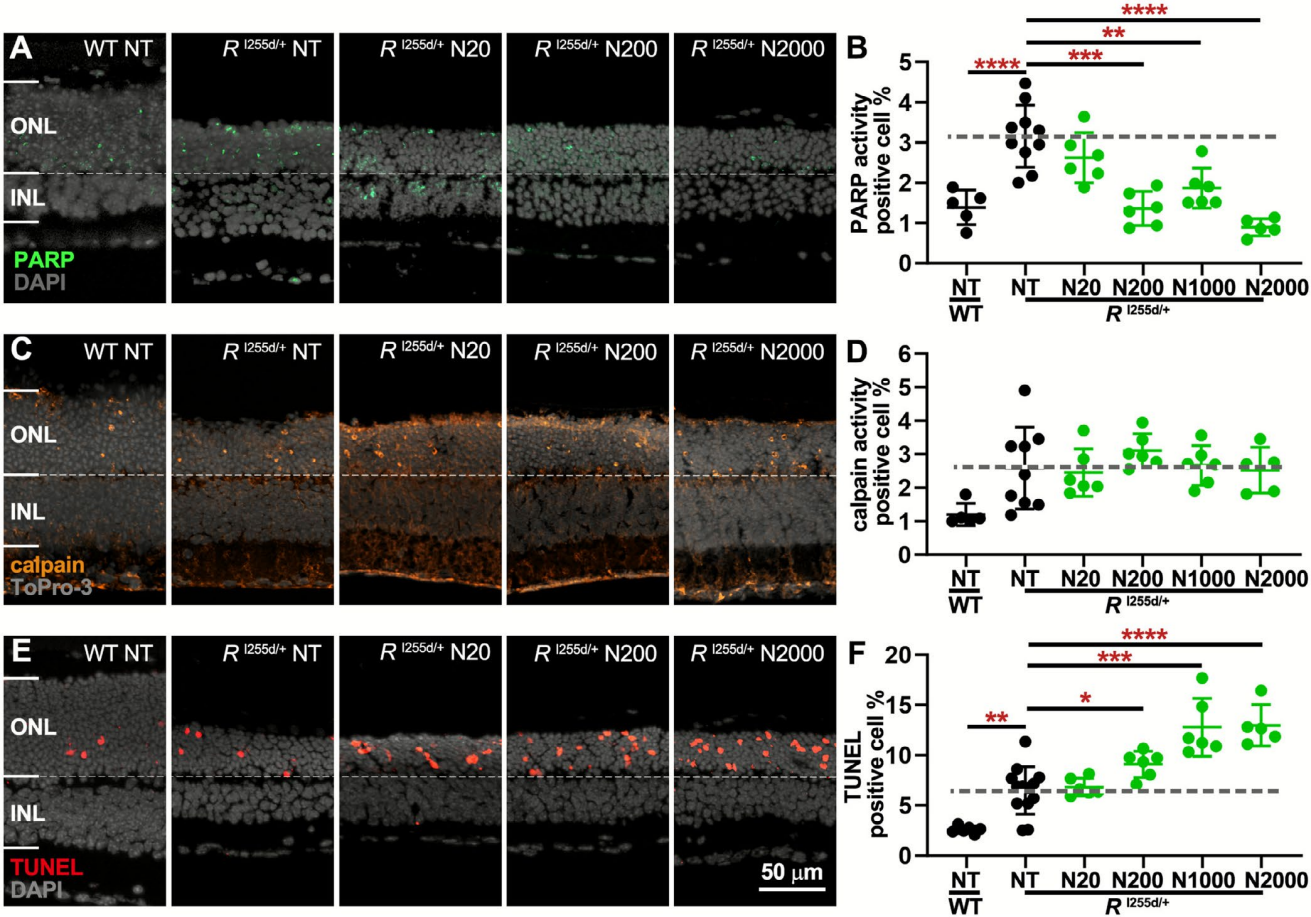
NAM decreases PARP activity, increases photoreceptor death. Organotypic retinal explants were derived from *Rho* ^I255d/+^ mice at post‐natal (P) day 12 and cultured with 20, 200, 1000, or 2000 μM of nicotinamide (NAM; N) from P14 to P20. Wild‐type (WT) data shown for comparison. (A, B) In mutant retina, PARP activity (green) was dose‐dependently decreased by NAM treatment. (C, D) Calpain activity (orange) in *Rho* ^I255d/+^ ONL was not changed by NAM. (E, F) ONL cell death (TUNEL assay, red) was dose‐dependently increased by NAM treatment. DAPI (A, E) and ToPro‐3 (C) were used as nuclear counterstains (gray). Data represents mean and SD, and were obtained from 5 to 13 independent retinal explant cultures; statistical analysis: Two‐way ANOVA with Dunnett's multiple comparisons test; * = *p* ≤ 0.05; ** = *p* ≤ 0.01; *** = *p* ≤ 0.001; **** = *p* ≤ 0.0001; INL = inner nuclear layer. Scale bar = 50 μm.

The PARP inhibitors Ola, Sar, and INO all exhibit some degree of DNA‐trapping, that is, they lock the PARP protein onto the DNA, impeding gene transcription or repair of DNA damage (Murai et al. 2012; Pires et al. 2025). To ascertain that the toxicity of PARP inhibition was not caused by DNA‐trapping and to ultimately settle the negative effects of PARP inhibition on photoreceptor viability in the *Rho* ^I255del/+^ mutant situation, we employed NAM as a classical inhibitor of PARP activity (Clark et al. 1971) that only blocks catalytic activity but does not cause DNA‐trapping. Using the above established test paradigm, we found NAM to also reduce PARP activity in *Rho* ^I255d/+^ ONL (Figure 6A,B, Table S8), albeit at much higher concentrations than the other inhibitors used in this study, in line with NAM's relatively high IC_50_ value (*cf*. Table 1). While calpain activity was not changed by NAM treatment (Figure 6C,D), the numbers of dying cells in the ONL were markedly and highly significantly increased at NAM concentrations of 200 μM or higher (Figure 6E,F). This data conclusively showed that in *Rho* ^I255d/+^ retina, PARP inhibition was strongly detrimental to rod photoreceptors.

### 3.7 Olaparib Shows the Strongest Detrimental Effects on *Rho* ^I255d^ ^/+^ Retina

**FIGURE 7:**
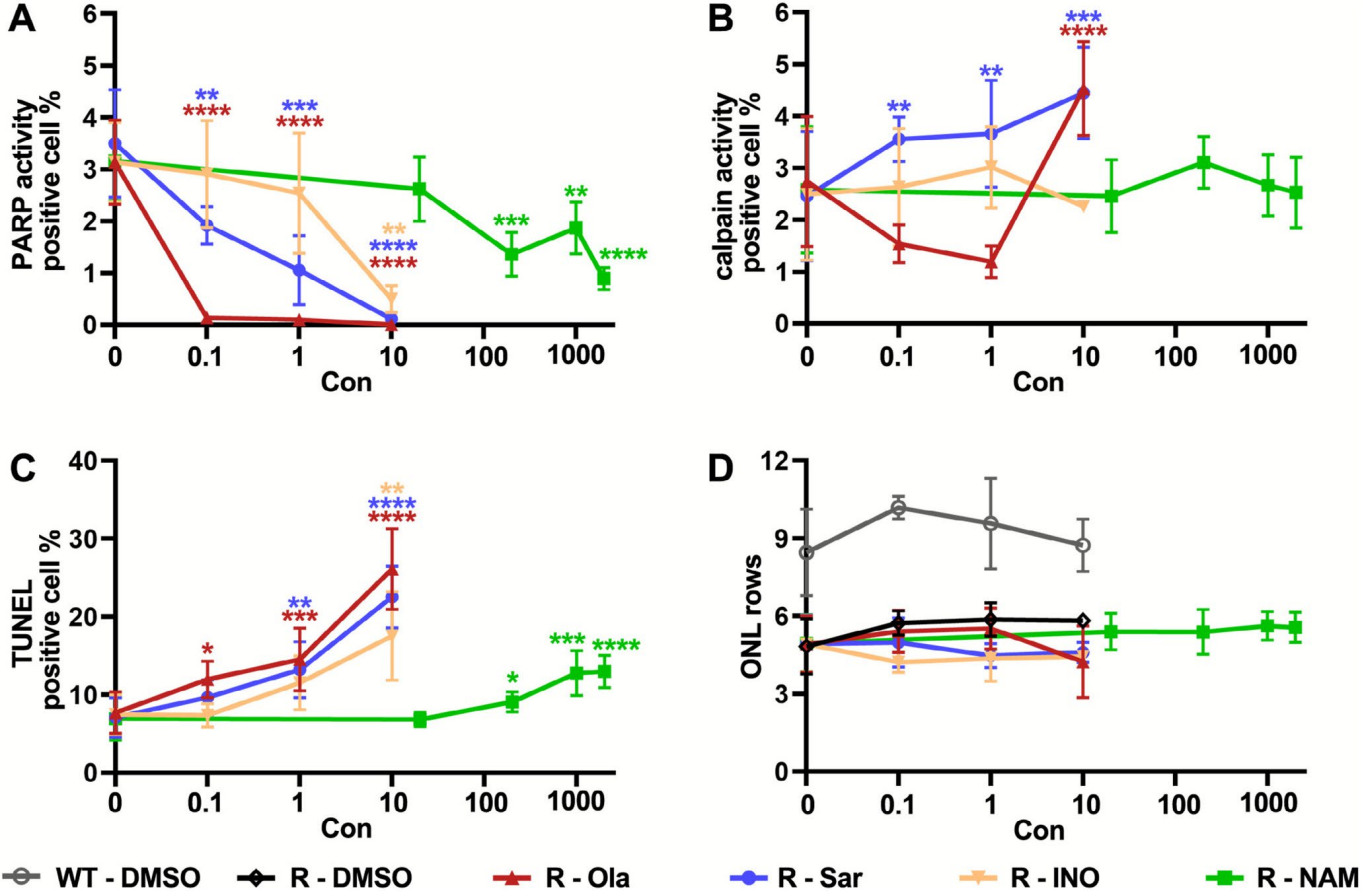
Inhibitors dose‐dependently reduce PARP activity and increase *Rho* ^I255d/+^ photoreceptor death. Summary graphs for the effects of treatment with olaparib (Ola, dark red), saruparib (Sar, blue), INO1001 (INO, light brown), and nicotinamide (NAM, green). DMSO (gray/black) shown as control. (A) The number of ONL cells displaying high PARP activity were reduced by all PARP inhibitors in a dose‐dependent manner, with Ola showing the strongest and NAM the weakest effect. (B) ONL calpain activity was increased by Sar treatment. (C) All four PARP inhibitors dose‐dependently increased ONL cell death. (D) In WT retina and *Rho* ^I255del/+^ retina, DMSO did not significantly change the ONL cell row counts. In mutant retina PARP ONL row counts were not affected by PARP inhibitors within the experimental time frame (post‐natal day 20). Error bars indicate SD; statistical analysis: One‐way and Two‐way ANOVA with Dunnett's multiple comparisons test; * = *p* ≤ 0.05; ** = *p* ≤ 0.01; *** = *p* ≤ 0.001; **** = *p* ≤ 0.0001.

To compare the effects of the four PARP inhibitors tested (Ola, Sar, INO, and NAM; Table 1), we plotted the combined data on PARP activity, calpain activity, cell death, and ONL row counts in summary graphs (Figure 7, Table S9). This comparison revealed that Ola had the strongest effect on PARP activity in the *Rho* ^I255d/+^ ONL and produced the most marked increase in ONL cell death. Conversely, NAM had the overall weakest effect on these parameters. Sar caused a strong rise in calpain activity already at relatively low concentrations, an effect that seemed disproportional to PARP inhibition, and which could indicate further off‐target effects of Sar. The number of ONL rows was not significantly affected by any of the PARP inhibitors used here, within the timeframe of the experiment. Yet, it seems likely that the increased cell death rates would have translated into stronger loss of ONL row counts if culturing had been prolonged by a few more days.

## 4 Discussion

Elevated PARP activity has been found in degenerating photoreceptors in a high number of animal models for RP and other inherited retinal degenerations (Arango‐Gonzalez et al. 2014), including in the recently generated *Rho* ^I255d^ autosomal dominant RP mouse (Cao, Dahlen, et al. 2024; Cao, Zhu, et al. 2024). This finding suggested using over‐activated PARP as a common target for therapeutic interventions in a large group of highly heterogeneous genetic diseases (Verbakel et al. 2018). Our present study, however, provides evidence that in the photoreceptors of *Rho* ^I255d/+^ autosomal‐dominant rhodopsin mutants, PARP activity is beneficial and required, so that its inhibition rather aggravates the disease phenotype. This highlights the need for a more comprehensive insight into underlying disease mechanisms to guide effective and safe future therapy developments.

### 4.1 A Novel, Apparently Ambiguous Role of PARP in Neurodegenerative Diseases

A property of PARP enzymes is to sense DNA damage, which triggers poly(ADP‐ribosyl)ation, a process in which PARP transfers ADP‐ribose moieties from NAD^+^ to target proteins, like the histones of the chromatin (Curtin and Szabo 2020). Such an activity is crucial for DNA repair; PARP has been referred to as the “guardian of the genome” (Chatterjee et al. 1999). The protective role of PARP was the basis for the development of PARP inhibitors as anti‐cancer drugs, which, especially when administered in combination with DNA‐damaging compounds or radiation, promote the death of mitotically active cells (Bhamidipati et al. 2023). This contrasts with excessive PARP activation frequently observed in neurodegenerative diseases, which has led to the concept of PARP‐mediated cell death or PARthanatos (Yang et al. 2024). Accordingly, PARP inhibitors originally developed for cancer therapy may serve as possible neuroprotective agents. In murine models for autosomal‐recessive RP of the retina, carrying homozygous disease‐causing mutations in *Pde6a* and *Pde6b* genes, inhibition of PARP with for example, PJ34 or Ola was found to significantly delay photoreceptor degeneration (Dong et al. 2023; Jiao et al. 2016; Paquet‐Durand et al. 2007; Sahaboglu et al. 2016; Yan et al. 2022). The protective effect of PARP inhibition has been attributed to a decreased consumption of NAD^+^, which is a critical cofactor in ATP‐producing energy metabolism. According to this hypothesis, overactivation of PARP may severely impair the capacity of a cell to produce ATP (Du et al. 2003), while, in contrast, PARP inhibition allows the maintenance of cellular ATP at essential levels. In different animal models for recessive and dominant RP, the levels of PARP activity at the respective peak of degeneration may present differently and may affect around 5% of ONL cells in the *Pde6b*‐mutant *rd1* mouse (Paquet‐Durand et al. 2007), around 2% in various *Pde6a*‐mutant mice (Jiao et al. 2016), and about 3%–4% in the *Rho* ^I255d/+^ ONL (Cao, Dahlen, et al. 2024; Cao, Zhu, et al. 2024).

The work presented here was obtained in the *Rho* ^I255d/+^ model of autosomal‐dominant RP, carrying one normal and one mutated allele, is in contradiction with earlier results derived from homozygous autosomal‐recessive RP models. The cell death‐promoting effects of the four different PARP inhibitors used in the present study unmistakably demonstrate that in *Rho* ^I255d/+^ photoreceptors PARP activity assumes a protective rather than a destructive role. As the main difference between autosomal dominant and autosomal recessive RP is the presence of a regular, unaffected allele in the latter, a reason for the findings may be that PARP inhibition may have differential effects on gene activation, transcription, and protein translation between diseased and normal alleles. Alternatively, the damaging effects of PARP inhibitors in a rhodopsin mutant may relate to the relatively high transcription rate of the *RHO* (DesJardin et al. 1993), since PARP activity is required for the initiation of gene transcription (Kraus and Hottiger 2013). Still, we did not observe changes in photoreceptor rhodopsin distribution or trafficking under Ola treatment. As in all autosomal‐dominant forms of RP, the I255del mutation is expected to lead to misfolding and aggregation of irregular rhodopsin protein in the endoplasmic reticulum (ER), and the ensuing unfolded protein response (UPR) may consume large amounts of ATP (Cao, Dahlen, et al. 2024; Cao, Zhu, et al. 2024) that would not be correctable by PARP inhibition. Rather, a sufficient degree of PARP activity may be required for the proper degradation of misfolded rhodopsin, since at least one PARP isoform—PARP16—was found to be a key regulator of UPR (Jwa and Chang 2012). Thus, it is plausible to think that PARP—or specific PARP isoforms—may be required to uphold adequate UPR function, and that in a condition of strong unfolded/misfolded protein overload, an inhibition of PARP is detrimental. This hypothesis would explain why PARP inhibition is beneficial in autosomal‐recessive RP forms where protein misfolding and UPR are usually not critical, and where reduced consumption of NAD^+^ and reduced strain on energy metabolism would improve cellular survival. Furthermore, *Rho* ^I255d/+^ cone photoreceptors would not be affected by a possible (mis‐)regulation of UPR by PARP inhibitors due to different opsin types, expression rates, and transport mechanisms.

Alternatively, it is possible that the detrimental effects of PARP inhibitors observed here could be due to off‐target effects on other proteins. For instance, Ola has been shown to affect certain kinase‐signaling pathways, such as ATM/ATR (Lloyd et al. 2020), CHK1 (Do et al. 2021), EGFR/Akt/p38/ERK (Chowdhury et al. 2019), or LcK and Fyn (Antolin et al. 2020), but may also affect drug transporters localized mainly to liver and kidney (MDR1, OCT1, OCT2, OATP1B1, OAT3, MATE1, and MATE2K) (McCormick and Swaisland 2017). However, Ola treatment did not negatively affect photoreceptor viability in the *rd1* mouse model for autosomal recessive RP (Sahaboglu et al. 2016; Yan et al. 2022). Hence, the negative effect of Ola on photoreceptor viability seems unlikely to stem from off‐targets. Moreover, in WT retina treated with Ola, a clear photoreceptor toxicity was seen only at a concentration of 10 μM, while in *Rho* ^I255d/+^ retina, Ola was toxic already at 0.1 μM. This two log‐unit difference again points to target specific effects of Ola treatment.

### 4.2 PARP Inhibition: Switching From Non‐Apoptotic to Apoptotic Cell Death?

Photoreceptor loss is strongly linked to (excessive) PARP activity (Belhadj et al. 2021; Paquet‐Durand et al. 2007), however, the exact underlying cell death mechanisms and how PARP may be involved in these are still unclear. Other than to PARthanatos, excessive activation of PARP may be connected to further, alternative cell death pathways, including necroptosis and cGMP‐dependent cell death (Cao, Dahlen, et al. 2024; Cao, Zhu, et al. 2024; Power et al. 2020; Virág et al. 2013; Zhu, Kumar, et al. 2025; Zhu, Peiroten, et al. 2025). In animal models for recessive and dominant RP, including in the *Rho* ^I255d/+^ model (Zhu, Kumar, et al. 2025; Zhu, Peiroten, et al. 2025), photoreceptor degeneration has been connected specifically to excessive cGMP signaling and non‐apoptotic forms of cell death (Power et al. 2020). In addition, PARP has also been proposed to be involved in the execution of apoptosis, although here the links are far less clear (Virág et al. 2013), notably because apoptosis, as an active, ATP‐dependent process (Liu et al. 1996), cannot be executed when NAD^+^ has been depleted (by PARP). In other words, the inhibition of PARP and the maintenance of cellular NAD^+^ and ATP at levels sufficiently high to allow for the execution of apoptosis could promote apoptotic cell death. This corresponds to our observation of increased caspase‐3 activation—a prototypical marker for apoptosis—in *Rho* ^I255d/+^ photoreceptors treated with Ola, which is in line with earlier studies in other cellular systems (Sauriol et al. 2023; Zhao et al. 2019). Thus, the activity of PARP or the lack thereof in rod photoreceptors may be able to switch the execution of cell death from non‐apoptotic to apoptotic mechanisms. This may have a bearing on retinal development since developmental cell death in the early post‐natal retina is mostly driven by apoptosis (Young 1984). However, since we have not used PARP inhibitors earlier than P14, we cannot exclude the possibility that this could have affected retinal development.

### 4.3 Cross‐Talk Between PARP and Calpain

Apart from PARP, photoreceptor cell death is also strongly linked to calpain activity, a Ca^2+^‐dependent protease that plays important functions under physiological and pathological conditions in the retina and other organs (Arango‐Gonzalez et al. 2014). In degenerative retinal diseases, high calpain activity has been attributed to mutation‐dependent Ca^2+^‐influx, failures of energy metabolism, and lack of ATP‐dependent Ca^2+^‐extrusion (Das et al. 2022). In the *rd1* mouse model, inhibition of PARP blocked calpain activity, whereas the inhibition of calpain did not reduce PARP activity, suggesting that PARP activity was upstream of calpain (Yan et al. 2022). In the present study, we thus expected to see a progressive decrease in calpain activity with rising PARP inhibitor concentrations. However, what we observed instead was either no change or—when treated with Sar or high‐dose Ola—an increase in calpain activity.

While additional work is needed to explore this phenomenon further, we hypothesize that in *Rho* ^I255d/+^ photoreceptors, under conditions of misfolded protein overload and a block of UPR by PARP inhibitors, cells may invoke other proteolytic systems. In this context, we note that the endoplasmic reticulum (ER) is an important store for intracellular Ca^2+^, and a dysfunction induced by PARP inhibitors could lead to excessive Ca^2+^ release and subsequent calpain activation. Prolonged release of Ca^2+^ from the ER may also disrupt mitochondrial membrane potential (Tripathi and Chaube 2012), which would lead to ATP depletion as well as to additional Ca^2+^ release. Both processes would promote calpain activation even though the release from intracellular Ca^2+^ may not be strong enough to attain the micromolar Ca^2+^‐levels required to activate calpain‐2. This would thus explain why, under Ola treatment, the overall calpain activity was significantly increased, while calpain‐2 activation was not.

## 5 Conclusion

In this work, we assessed the impact of different PARP inhibitors on the progression of photoreceptor degeneration in a novel, human‐homologous rhodopsin‐mutant *Rho* ^I255del/+^ mouse model for autosomal dominant RP. So far, elevated PARP activity has been identified as a factor promoting neurodegeneration in animal models of autosomal recessive RP forms. Contrary to these studies performed mostly in *Pde6*‐mutant lines, the treatment data obtained here, using four different and well‐established compounds, conclusively demonstrate the destructive effects of PARP inhibition in the autosomal dominant rhodopsin‐mutant *Rho* ^I255del^ model. These detrimental effects may be due to the coexistence of regular and mutated alleles, a misregulation of the unfolded protein response, and/or a shift in degenerative mechanisms from cGMP‐dependent pathway to classical apoptosis. The latter option may be governed by PARP‐inhibition mediated improvements in photoreceptor energy metabolism. Our observations highlight the importance of carefully considering potential fields of application when utilizing PARP inhibition as a therapeutic strategy, especially in the context of retinal degeneration. Moreover, since PARP inhibitors were initially developed for anti‐cancer therapy, and since adverse effects were also observed in WT retina, the widespread use of compounds like olaparib may raise concerns about potential side effects on the retina.

## Author Contributions

**Yu Zhu:** conceptualization, methodology, software, data curation, investigation, validation, formal analysis, visualization, writing – original draft, writing – review and editing. **Azdah Hamed A. Fallatah:** methodology, investigation, writing – review and editing. **Kangwei Jiao:** writing – review and editing. **Mathias W. Seeliger:** writing – review and editing. **François Paquet‐Durand:** conceptualization, supervision, funding acquisition, project administration, writing – review and editing.

## Funding

This research was funded by grants from the Charlotte and Tistou Kerstan Foundation (RHO‐Cure, PD2017), Key Project of Yunnan Fundamental Research Projects (202301AS070046), and the ProRetina Foundation.

## Conflicts of Interest

The authors declare no conflicts of interest.

## Supporting information


**Figure S1:** Outer‐/Inner‐ segment length in *Rho* ^I255del/+^ retina after olaparib treatment.
**Figure S2:** Olaparib reduces PARP activity and triggers *Rho* ^I255d/+^ photoreceptor cell death already at P18.
**Table S1:** The effect of olaparib (Ola) on PARP activity (PARP in situ assay) and photoreceptor cell death (TUNEL) in WT and Rhol^255d/+^.
**Table S2:** Effect of olaparib (Ola) on calpain activity (calpain in situ assay), calpain‐2 (calpain‐2 staining) and caspase‐3 activation (caspase‐3 staining), rhodopsin (rhodopsin staining), and cone survival (cone arrestin‐3 staining) in outer nuclear layer (ONL) or/and inner nuclear layer (INL), with concentrations (0.1, 1, and 10 μM).
**Table S3:** Effect of olaparib (Ola) lasting 4 days on the length of cone inner and outer segments (IS/OS) by Figure S1.
**Table S4:** Effect of olaparib (Ola) lasting 4 days on the activity of PARP (PARP in situ assay) and calpain.
**Table S5:** Comparison of the Effect of 1 μM olaparib (Ola) lasting from post‐natal (P) Day 12 to P18 (4 days).
**Table S6:** Effect of saruparib (Sar) on the activity of PARP (PARP in situ assay) and calpain (calpain in situ assay).
**Table S7:** Effect of INO1001 (INO) on the activity of PARP (PARP in situ assay) and calpain (calpain in situ assay).
**Table S8:** Effect of nicotinamide (NAM) on the activity of PARP (PARP in situ assay) and calpain (calpain in situ assay).
**Table S9:** Effect of DMSO and PARP inhibitors, including olaparib (Ola), saruparib (Sar), INO1001 (INO), and nicotinamide (NAM), on outer nuclear layer (ONL) rows counts, with different concentrations, DMSO (0.01%, 0.1% and 1%), Ola, Sar and INO (0.1, 1, and 1 μM), and NAM (20, 200, 1000, and 2000 μM): Quantitative data for graphs presented in Figure 7D. Test = Shapiro–Wilk normality test, *P* = pass, *N* = Not pass.

## Data Availability

All data generated or analyzed during this study are included in this published article and its Supporting Information files.
