# Supplementary material for: PARP Activity Is Essential for Retinal Photoreceptor Survival in the Human Homologous Rho I255del Mouse Model for Autosomal Dominant Retinitis Pigmentosa
Source: J Neurochem. 2025 Dec 22;169(12):e70319. doi: 10.1111/jnc.70319 (PMC12721004; doi:10.1111/jnc.70319)
Supplement: Supplementary file 1 — Figure S1: Outer‐/Inner‐ segment length in Rho I255del/+ retina after olaparib treatment. Figure S2: Olaparib reduces PARP activity and triggers Rho I255d/+ photoreceptor cell death already at P18. Table S1: The effect of olaparib (Ola) on PARP activity (PARP in situ assay) and photoreceptor cell death (TUNEL) in WT and Rhol255d/+. Table S2: Effect of olaparib (Ola) on calpain activity (calpain in situ assay), calpain‐2 (calpain‐2 staining) and caspase‐3 activation (caspase‐3 staining), rhodopsin (rhodopsin staining), and cone survival (cone arrestin‐3 staining) in outer nuclear layer (ONL) or/and inner nuclear layer (INL), with concentrations (0.1, 1, and 10 μM). Table S3: Effect of olaparib (Ola) lasting 4 days on the length of cone inner and outer segments (IS/OS) by Figure S1. Table S4: Effect of olaparib (Ola) lasting 4 days on the activity of PARP (PARP in situ assay) and calpain. Table S5: Comparison of the Effect of 1 μM olaparib (Ola) lasting from post‐natal (P) Day 12 to P18 (4 days). Table S6: Effect of saruparib (Sar) on the activity of PARP (PARP in situ assay) and calpain (calpain in situ assay). Table S7: Effect of INO1001 (INO) on the activity of PARP (PARP in situ assay) and calpain (calpain in situ assay). Table S8: Effect of nicotinamide (NAM) on the activity of PARP (PARP in situ assay) and calpain (calpain in situ assay). Table S9: Effect of DMSO and PARP inhibitors, including olaparib (Ola), saruparib (Sar), INO1001 (INO), and nicotinamide (NAM), on outer nuclear layer (ONL) rows counts, with different concentrations, DMSO (0.01%, 0.1% and 1%), Ola, Sar and INO (0.1, 1, and 1 μM), and NAM (20, 200, 1000, and 2000 μM): Quantitative data for graphs presented in Figure 7D. Test = Shapiro–Wilk normality test, P = pass, N = Not pass. [file JNC-169-0-s001.pdf]

PARP activity is essential for retinal photoreceptor survival in the human homologous *Rho*^{I255del} mouse model for autosomal dominant retinitis pigmentosa

Authors: Yu Zhu[1,2], Azdah Hamed A Fallatah[1,2], Kangwei Jiao[3], Mathias W. Seeliger[4], François Paquet-Durand[1*]

Affiliations:

[1] Cell Death Mechanisms group, Institute for Ophthalmic Research, University of Tübingen, Germany

[2] Graduate School for Cellular and Molecular Neuroscience, University of Tübingen, Germany

[3] Key Laboratory of Yunnan Province, Yunnan Eye Institute, Affiliated Hospital of Yunnan University, Yunnan University, Kunming, 650021, Yunnan, China

[4] Division of Ocular Neurodegeneration, Institute for Ophthalmic Research, University of Tübingen, Germany

[*] to whom correspondence should be addressed: francois.paquet-durand@uni-tuebingen.de

Supplemental figures

Figure S1

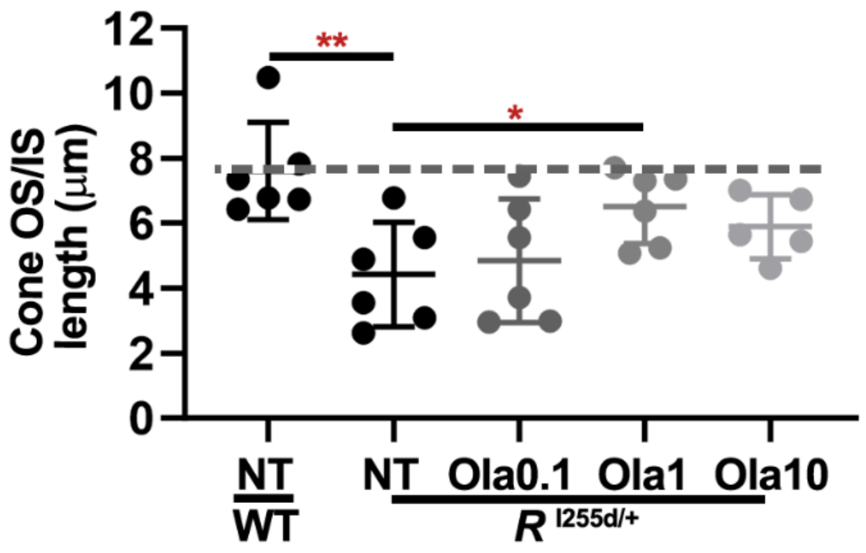

Figure S1. Outer- / inner- segment length in *Rho*$^{I255del/+}$ retina after olaparib treatment. Organotypic retinal explants derived from wild-type (WT) and *Rho*$^{I255del/+}$ mice were cultivated from postnatal (P) 12 to P20 with different olaparib (Ola) concentrations (0.1, 1, 10 µM). The combined length of outer- and inner-segments (OS/IS) was assessed based on cone arrestin-3 immunostaining. WT, non-treated (NT) retinas had significantly longer cone OS/IS than NT mutant retinas. In mutant retinas, treatment with Ola 1 µM significantly increased cone OS/IS length. Images are representative of results obtained in 5-6 independent retinal cultures; error bars indicate SD; statistical analysis: Two-way ANOVA with Dunnett's multiple comparisons test; * = $p \leq 0.05$; ** = $p \leq 0.01$.

Figure S2

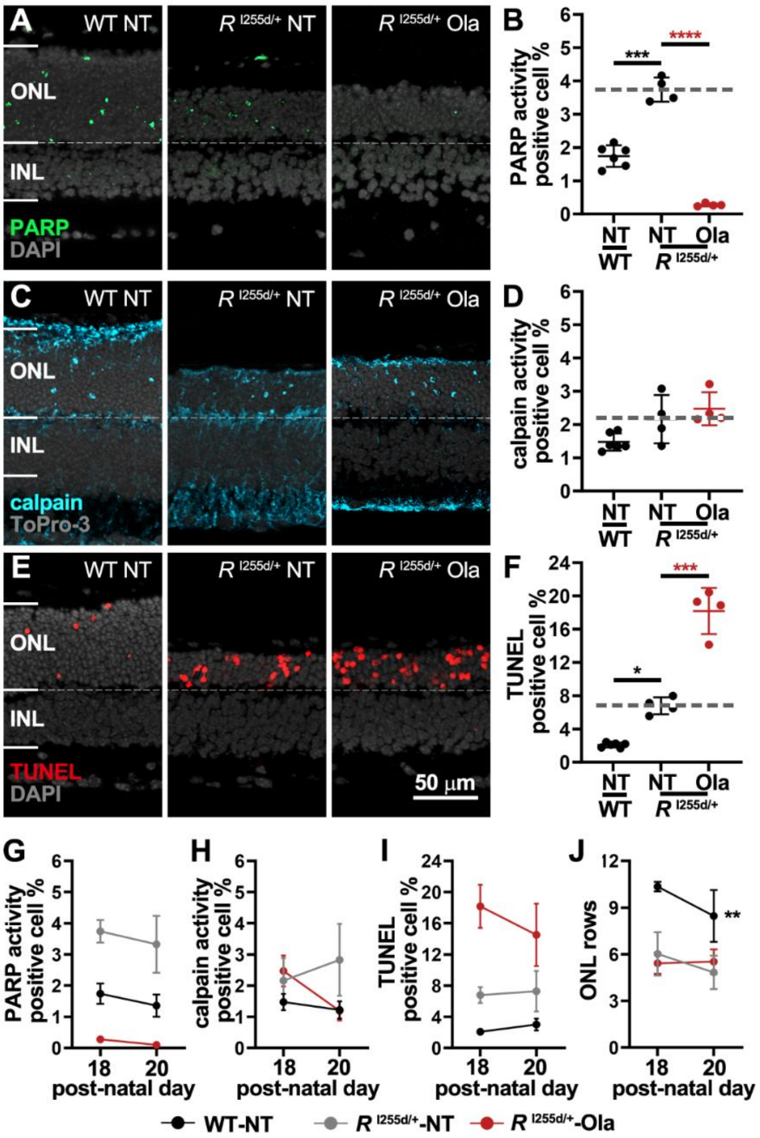

Figure S2. Olaparib reduces PARP activity and triggers *Rho*$^{I255d/+}$ photoreceptor cell death already at P18. Organotypic retinal explants were derived from wild-type (WT) and *Rho*$^{I255d/+}$ ($R^{I255d/+}$) mice and cultured from post-natal (P) day 12 to P18. Cultures were either non-treated (NT) or treated with 1 µM olaparib (Ola). (**A-B**) In P18 retina, PARP activity (green) was increased in the mutant compared to WT, but strongly decreased by Ola treatment. (**C-D**) The overall activity of calpain (cyan) in the *Rho*$^{I255d/+}$ mutant was not changed by Ola treatment. (**E-F**) Cell death in the outer nuclear layer (ONL) as indicated by the TUNEL assay (red), was significantly increased in mutant retina compared to WT. Cell death was strongly increased by Ola treatment. (**G**-**J**) Comparison of Ola treatment effects in retinal explants cultured until P18 and P20. (**G**) In both WT and mutant NT retina PARP activity decreased slightly from P18 to P20. (**H**) From P18 to P20 overall calpain activity increased in NT mutant retinas, and 1µM Ola treatment slightly reduced calpain activity in the mutant situation. (**I**) The TUNEL assay showed a minor increase in cell death in both NT WT and mutant retina from P18 to P20. In Ola-treated retina, cell death appeared to be reduced. (**G**) The number of ONL rows, as an independent marker for photoreceptor survival, decreased in NT WT. Ola treatment did not significantly improve photoreceptor survival. DAPI (**A**, **E**) and ToPro-3 (**C**) were used as nuclear counterstains (grey). Images are representative of results obtained in 3-20 independent retinal cultures; error bars indicate SD; statistical analysis: Two-way ANOVA with Dunnett's multiple comparisons test; * = $p \leqslant 0.05$; ** = $p \leqslant 0.01$; *** = $p \leqslant 0.001$; **** = $p \leqslant 0.0001$; * on the top: comparison of NT at same timepoint; * on the right: comparison of P20 with P18. INL = inner nuclear layer. Scale bar = 50 µm.

Supplemental Tables

Table S1. The effect of olaparib (Ola) on PARP activity (PARP *in situ* assay) and photoreceptor cell death (TUNEL) in WT and *Rho*$^{I255d/+}$: Quantitative data for graphs presented in Figure 2 C, D, G, H. Test = Shapiro-Wilk normality test, P = pass, N = Not pass.

Table S1	Parameter	*p* - value comparison	Genotype-treatment	Mean ± SD (%)	*p* - value	n	DF (column)	F – value	*p* - value	Test
Fig. 2C	PARP activity	WT - NT	WT - NT	1.36 ± 0.36		7	Between (6) Within (22)	F (6, 22) = 20.72	*p* < 0.0001	P
			WT - D 0.01	1.23 ± 0.27	*p* = 0.9383	4				P
			WT - D 0.1	0.92 ± 0.09	*p* = 0.1042	3				P
			WT - D 1	1.31 ± 0.26	*p* = 0.9996	4				P
			WT - Ola 0.1	0.39 ± 0.25	*p* < 0.0001	4				P
			WT - Ola 1	0.05 ± 0.04	*p* < 0.0001	3				P
			WT - Ola 10	0.11 ± 0.13	*p* < 0.0001	4				P
Fig. 2D		$R^{I255d/+}$ - NT	$R^{I255d/+}$ - NT	3.45 ± 0.93		10	Between (6) Within (23)	F (6, 23) = 33.07	*p* < 0.0001	P
			$R^{I255d/+}$ - D 0.01	3.85 ± 0.65	*p* = 0.8792	3				N
			$R^{I255d/+}$ - D 0.1	2.78 ± 0.38	*p* = 0.4733	3				P
			$R^{I255d/+}$ - D 1	3.04 ± 0.08	*p* = 0.8723	3				P
			$R^{I255d/+}$ - Ola 0.1	0.13 ± 0.03	*p* < 0.0001	4				P
			$R^{I255d/+}$ - Ola 1	0.10 ± 0.02	*p* < 0.0001	3				P
			$R^{I255d/+}$ - Ola 10	0.00 ± 0.00	*p* < 0.0001	4				N
Fig. 2G	TUNEL	WT - NT	WT - NT	3.03 ± 0.77		10	Between (6) Within (26)	F (6, 26) = 65.48	*p* < 0.0001	P
			WT - D 0.01	2.01 ± 0.81	*p* = 0.1498	4				P
			WT - D 0.1	1.81 ± 0.78	*p* = 0.1041	3				P
			WT - D 1	2.59 ± 0.36	*p* = 0.8792	4				N
			WT - Ola 0.1	3.10 ± 0.77	*p* > 0.9999	4				P
			WT - Ola 1	3.63 ± 1.06	*p* = 0.6479	4				P
			WT - Ola 10	10.57 ± 0.46	*p* < 0.0001	4				P
Fig. 2H		$R^{I255d/+}$ - NT	$R^{I255d/+}$ - NT	7.68 ± 2.70		14	Between (6) Within (40)	F (6, 40) = 26.96	*p* < 0.0001	P
			$R^{I255d/+}$ - D 0.01	7.12 ± 3.28	*p* = 0.9999	3				P
			$R^{I255d/+}$ - D 0.1	5.96 ± 0.86	*p* = 0.9445	3				P
			$R^{I255d/+}$ - D 1	7.69 ± 1.81	*p* > 0.9999	3				P
			$R^{I255d/+}$ - Ola 0.1	11.95 ± 2.35	*p* = 0.0232	9				P
			$R^{I255d/+}$ - Ola 1	14.51 ± 4.02	*p* = 0.0001	9				P
			$R^{I255d/+}$ - Ola 10	26.11 ± 5.20	*p* < 0.0001	6				P

Table S2. Effect of olaparib (Ola) on calpain activity (calpain *in situ* assay), calpain-2 (calpain-2 staining) and caspase-3 activation (caspase-3 staining), rhodopsin (rhodopsin staining), and cone survival (cone arrestin-3 staining) in outer nuclear layer (ONL) or/and inner nuclear layer (INL), with concentrations (0.1, 1, and 10 µM): Quantitative data for graphs presented in Figures 3B, D, F, H, J. Test = Shapiro-Wilk normality test, P = pass, N = Not pass.

Table S2	Parameter	*p* - value comparison	Genotype-treatment	Mean ± SD (%)	*p* - value	n	DF (column)	F – value	*p* - value	Test
Fig. 3B	calpain activity	$R^{I255d/+}$ - NT	WT - NT	1.22 ± 0.27	$p = 0.0184$	7	Between (4) Residual (13)	F (4, 13) = 26.65	$p < 0.0001$	N
			$R^{I255d/+}$ - NT	2.74 ± 1.26		10				P
			$R^{I255d/+}$ - Ola 0.1	1.54 ± 0.37	$p = 0.6430$	4				P
			$R^{I255d/+}$ - Ola 1	1.19 ± 0.31	$p = 0.3048$	3				P
			$R^{I255d/+}$ - Ola 10	4.53 ± 0.90	$p < 0.0001$	4				P
Fig. 3D	calpain-2	$R^{I255d/+}$ - NT	WT - NT	1.31 ± 0.74	$p = 0.0126$	3	Between (4) Residual (14)	F (4, 14) = 5.633	$p = 0.0065$	P
			$R^{I255d/+}$ - NT	4.38 ± 0.46		3				P
			$R^{I255d/+}$ - Ola 0.1	2.96 ± 0.72	$p = 0.0968$	8				P
			$R^{I255d/+}$ - Ola 1	3.21 ± 0.75	$p = 0.1463$	6				P
			$R^{I255d/+}$ - Ola 10	4.88 ± 1.95	$p > 0.9999$	6				P
Fig. 3F	caspase-3	$R^{I255d/+}$ - NT	WT - NT	0.25 ± 0.09	$p = 0.0208$	3	Between (4) Residual (14)	F (4, 14) = 14.74	$p < 0.0001$	P
			$R^{I255d/+}$ - NT	2.52 ± 0.78		6				P
			$R^{I255d/+}$ - Ola 0.1	2.22 ± 0.52	$p = 0.6210$	7				P
			$R^{I255d/+}$ - Ola 1	3.96 ± 1.00	$p = 0.0371$	6				P
			$R^{I255d/+}$ - Ola 10	4.76 ± 1.52	$p = 0.0331$	6				P
Fig. 3H	rhodopsin	$R^{I255d/+}$ - NT	WT - NT	1.99 ± 0.21	$p < 0.0001$	7	Between (4) Residual (22)	F (4, 22) = 41.26	$p < 0.0001$	P
			$R^{I255d/+}$ - NT	8.18 ± 1.23		9				P
			$R^{I255d/+}$ - Ola 0.1	8.45 ± 0.44	$p = 0.9570$	6				P
			$R^{I255d/+}$ - Ola 1	8.37 ± 1.62	$p = 0.9827$	6				P
			$R^{I255d/+}$ - Ola 10	8.17 ± 1.37	$p > 0.9999$	7				P
Fig. 3J	cone arrestin-3	$R^{I255d/+}$ - NT	WT - NT	16.74 ± 1.99	$p = 0.0067$	6	Between (4) Residual (19)	F (4, 19) = 4.801	$p = 0.0076$	N
			$R^{I255d/+}$ - NT	12.32 ± 1.82		6				P
			$R^{I255d/+}$ - Ola 0.1	13.52 ± 2.80	$p = 0.7353$	6				P
			$R^{I255d/+}$ - Ola 1	12.91 ± 1.71	$p = 0.9686$	6				P
			$R^{I255d/+}$ - Ola 10	11.81 ± 1.52	$p = 0.9866$	5				P

Table S3. Effect of olaparib (Ola) lasting 4 days on the length of cone inner and outer segments (IS/OS) by immunostaining on cone arrestin-3, with concentrations (0.1, 1, and 10 µM): Quantitative data for graphs presented in Figure S1. Test = Shapiro-Wilk normality test, P = pass, N = Not pass.

Table S3	Parameter	*p* - value comparison	Genotype-treatment	Mean ± SD (%)	*p* - value	n	DF (column)	F – value	*p* - value	Test
Fig. S1	Cone IS/OS length	$R^{I255d/+}$ - NT	WT - NT	7.60 ± 1.50	$p = 0.0019$	6	Between (4) Residual (19)	F (4, 19) = 5.622	$p = 0.0037$	N
			$R^{I255d/+}$ - NT	4.42 ± 1.60		6				P
			$R^{I255d/+}$ - Ola 0.1	4.84 ± 1.90	$p = 0.9461$	6				P
			$R^{I255d/+}$ - Ola 1	6.51 ± 1.14	$p = 0.0431$	6				P
			$R^{I255d/+}$ - Ola 10	5.89 ± 0.98	$p = 0.2950$	5				P

Table S4. Effect of olaparib (Ola) lasting 4 days on the activity of PARP (PARP *in situ* assay) and calpain (calpain *in situ* assay), photoreceptor cell death (TUNEL) in outer nuclear layer (ONL) with concentration (1 µM): Quantitative data for graphs presented in Figures S2B, D, F. Test = Shapiro-Wilk normality test, P = pass, N = Not pass.

Table S4	Parameter	*p* - value comparison	Genotype-treatment	Mean ± SD (%)	*p* - value	n	DF (column)	F – value	*p* - value	Test
Fig. S2B	PARP activity	$R^{I255d/+}$ - NT	WT - NT	1.74 ± 0.33	$p = 0.0002$	6	Between (2) Residual (6)	F (2, 6) = 154.9	$p < 0.0001$	P
			$R^{I255d/+}$ - NT	3.74 ± 0.36		4				P
			$R^{I255d/+}$ - Ola 1	0.27 ± 0.05	$p < 0.0001$	4				P
Fig. S2D	calpain activity	$R^{I255d/+}$ - NT	WT - NT	1.47 ± 0.26	$p = 0.1040$	6	Between (2) Residual (6)	F (2, 6) = 6.156	$p = 0.0352$	P
			$R^{I255d/+}$ - NT	2.16 ± 0.73		4				P
			$R^{I255d/+}$ - Ola 1	2.47 ± 0.50	$p = 0.4466$	4				N
Fig. S2F	TUNEL	$R^{I255d/+}$ - NT	WT - NT	2.07 ± 0.29	$p = 0.0227$	6	Between (2) Residual (6)	F (2, 6) = 72.14	$p < 0.0001$	P
			$R^{I255d/+}$ - NT	6.79 ± 1.02		4				P
			$R^{I255d/+}$ - Ola 1	18.18 ± 2.78	$p = 0.0003$	4				P

Table S5. Comparison of the Effect of 1 µM olaparib (Ola) lasting from post-natal (P) day 12 to P18 (4 days) and P20 (6 days) on the activity of PARP (PARP *in situ* assay) and calpain (calpain *in situ* assay), photoreceptor cell death (TUNEL) in outer nuclear layer (ONL), and ONL rows count: Quantitative data for graphs presented in Figures S2G, H, I, J. Test = Shapiro-Wilk normality test, P = pass, N = Not pass.

Table S5	Parameter	Genotype-treatment	Endpoint	Mean ± SD (%)	*p* - value compare to P18	n	DF (row)	F – value	*p* - value	Test
Fig. S2G	PARP activity	WT-NT	P18	1.74 ± 0.33		6	Interaction (2) Row (1) Column (2) Residual (30)	Row-F (1, 30) = 2.098	*p* = 0.1579	P
			P20	1.36 ± 0.36	*p* = 0.6081	7				P
		$R^{I255d/+}$ - NT	P18	3.74 ± 0.36		4				P
			P20	3.33 ± 0.91	*p* = 0.5715	12				P
		$R^{I255d/+}$ - Ola 1	P18	0.27 ± 0.05		4				P
			P20	0.10 ± 0.02	*p* = 0.9740	3				P
Fig. S2H	calpain activity	WT-NT	P18	1.47 ± 0.26		6	Interaction (2) Row (1) Column (2) Residual (30)	Row-F (1, 30) = 1.024	*p* = 0.3197	P
			P20	1.22 ± 0.27	*p* = 0.9171	7				N
		$R^{I255d/+}$ - NT	P18	2.16 ± 0.73		4				P
			P20	2.83 ± 1.16	*p* = 0.3788	12				P
		$R^{I255d/+}$ - Ola 1	P18	2.47 ± 0.50		4				N
			P20	1.19 ± 0.31	*p* = 0.1103	3				P
Fig. S2I	TUNEL	WT-NT	P18	2.07 ± 0.29		6	Interaction (2) Row (1) Column (2) Residual (47)	Row-F (1, 47) = 0.8654	*p* = 0.3570	P
			P20	3.03 ± 0.77	*p* = 0.8426	10				P
		$R^{I255d/+}$ - NT	P18	6.79 ± 1.02		4				P
			P20	7.28 ± 2.60	*p* = 0.9778	20				P
		$R^{I255d/+}$ - Ola 1	P18	18.18 ± 2.78		4				P
			P20	14.51 ± 4.02	*p* = 0.0518	9				P
Fig. S2J	ONL rows	WT-NT	P18	10.34 ± 0.31		6	Interaction (2) Row (1) Column (2) Residual (42)	Row-F (1, 42) = 7.418	*p* = 0.0094	P
			P20	8.46 ± 1.66	*p* = 0.0076	9				P
		$R^{I255d/+}$ - NT	P18	6.02 ± 1.39		4				P
			P20	4.83 ± 1.07	*p* = 0.1810	16				P
		$R^{I255d/+}$ - Ola 1	P18	5.42 ± 0.69		4				P
			P20	5.53 ± 0.80	*p* = 0.9979	9				P

Table S6. Effect of saruparib (Sar) on the activity of PARP (PARP *in situ* assay) and calpain (calpain *in situ* assay), photoreceptor cell death (TUNEL) in outer nuclear layer (ONL), with concentrations (0.1, 1, and 10 µM): Quantitative data for graphs presented in Figures 4B, D, F. Test = Shapiro-Wilk normality test, P = pass, N = Not pass.

Table S6	Parameter	*p* - value comparison	Genotype-treatment	Mean ± SD (%)	*p* - value	n	DF (column)	F – value	*p* - value	Test
Fig. 4B	PARP activity	$R^{I255d/+}$ - NT	WT - NT	1.28 ± 0.34	*p* = 0.0004	5	Between (4) Residual (15)	F (4, 15) = 17.47	*p* < 0.0001	P
			$R^{I255d/+}$ - NT	3.50 ± 1.04		8				P
			$R^{I255d/+}$ - Sar 0.1	1.92 ± 0.36	*p* = 0.0094	5				P
			$R^{I255d/+}$ - Sar 1	1.05 ± 0.67	*p* = 0.0001	5				N
			$R^{I255d/+}$ - Sar 10	0.11 ± 0.09	*p* < 0.0001	4				P
Fig. 4D	calpain activity	$R^{I255d/+}$ - NT	WT - NT	1.10 ± 0.10	*p* = 0.5301	5	Between (4) Residual (15)	F (4, 15) = 19.81	*p* < 0.0001	P
			$R^{I255d/+}$ - NT	2.46 ± 1.25		8				P
			$R^{I255d/+}$ - Sar 0.1	3.56 ± 0.43	*p* = 0.0024	5				P
			$R^{I255d/+}$ - Sar 1	3.66 ± 1.03	*p* = 0.0015	5				P
			$R^{I255d/+}$ - Sar 10	4.45 ± 0.88	*p* = 0.0001	4				P
Fig. 4F	TUNEL	$R^{I255d/+}$ - NT	WT - NT	2.91 ± 0.86	*p* = 0.0651	6	Between (4) Residual (16)	F (4, 16) = 39.99	*p* < 0.0001	P
			$R^{I255d/+}$ - NT	7.07 ± 2.55		14				P
			$R^{I255d/+}$ - Sar 0.1	9.67 ± 2.23	*p* = 0.2404	5				P
			$R^{I255d/+}$ - Sar 1	13.21 ± 3.61	*p* = 0.0023	5				P
			$R^{I255d/+}$ - Sar 10	22.52 ± 3.98	*p* < 0.0001	4				P

Table S7. Effect of INO1001 (INO) on the activity of PARP (PARP *in situ* assay) and calpain (calpain *in situ* assay), photoreceptor cell death (TUNEL) in outer nuclear layer (ONL), with concentrations (0.1, 1, and 10 µM): Quantitative data for graphs presented in Figures 5B, D, F. Test = Shapiro-Wilk normality test, P = pass, N = Not pass.

Table S7	Parameter	*p* - value comparison	Genotype-treatment	Mean ± SD (%)	*p* - value	n	DF (column)	F – value	*p* - value	Test
Fig. 5B	PARP activity	$R^{I255d/+}$ - NT	WT - NT	1.26 ± 0.33	*p* = 0.0045	5	Between (4) Residual (14)	F (4, 14) = 8.571	*p* = 0.0010	P
			$R^{I255d/+}$ - NT	3.14 ± 0.77		10				P
			$R^{I255d/+}$ - INO 0.1	2.91 ± 1.03	*p* = 0.7807	6				P
			$R^{I255d/+}$ - INO 1	2.54 ± 1.16	*p* = 0.6953	4				P
			$R^{I255d/+}$ - INO 10	0.50 ± 0.26	*p* = 0.0011	3				P
Fig. 5D	calpain activity	$R^{I255d/+}$ - NT	WT - NT	1.10 ± 0.11	*p* = 0.3810	5	Between (4) Residual (14)	F (4, 14) = 2.655	*p* = 0.0773	P
			$R^{I255d/+}$ - NT	2.49 ± 1.27		8				P
			$R^{I255d/+}$ - INO 0.1	2.63 ± 1.13	*p* = 0.9033	6				P
			$R^{I255d/+}$ - INO 1	3.02 ± 0.78	*p* = 0.3447	4				P
			$R^{I255d/+}$ - INO 10	2.26 ± 0.10	*p* = 0.9458	3				P
Fig. 5F	TUNEL	$R^{I255d/+}$ - NT	WT - NT	2.91 ± 0.76	*p* = 0.0951	6	Between (4) Residual (15)	F (4, 15) = 11.42	*p* = 0.0002	P
			$R^{I255d/+}$ - NT	7.46 ± 2.53		14				P
			$R^{I255d/+}$ - INO 0.1	7.35 ± 1.50	*p* = 0.9998	6				N
			$R^{I255d/+}$ - INO 1	11.52 ± 3.45	*p* = 0.1738	4				P
			$R^{I255d/+}$ - INO 10	17.54 ± 5.69	*p* = 0.0013	3				P

Table S8. Effect of nicotinamide (NAM) on the activity of PARP (PARP *in situ* assay) and calpain (calpain *in situ* assay), photoreceptor cell death (TUNEL) in outer nuclear layer (ONL), with concentrations (20, 200, 1000 and 2000 µM): Quantitative data for graphs presented in Figures 6B, D, F. Test = Shapiro-Wilk normality test, P = pass, N = Not pass.

Table S8	Parameter	*p* - value comparison	Genotype-treatment	Mean ± SD (%)	*p* - value	n	DF (column)	F – value	*p* - value	Test
Fig. 6B	PARP activity	$R^{I255d/+}$ - NT	WT - NT	1.39 ± 0.43	$p < 0.0001$	5	Between (5) Residual (19)	F (5, 19) = 18.02	$p < 0.0001$	P
			$R^{I255d/+}$ - NT	3.16 ± 0.77		10				P
			$R^{I255d/+}$ - NAM 20	2.62 ± 0.62	$p = 0.0972$	6				P
			$R^{I255d/+}$ - NAM 200	1.36 ± 0.42	$p = 0.0001$	6				P
			$R^{I255d/+}$ - NAM 1000	1.87 ± 0.50	$p = 0.0017$	6				P
			$R^{I255d/+}$ - NAM 2000	0.89 ± 0.21	$p < 0.0001$	5				P
Fig. 6D	calpain activity	$R^{I255d/+}$ - NT	WT - NT	1.21 ± 0.33	$p = 0.3754$	5	Between (5) Residual (19)	F (5, 19) = 1.12	$p = 0.3832$	N
			$R^{I255d/+}$ - NT	2.58 ± 1.22		9				P
			$R^{I255d/+}$ - NAM 20	2.46 ± 0.71	$p = 0.9444$	6				P
			$R^{I255d/+}$ - NAM 200	3.11 ± 0.50	$p = 0.9949$	6				P
			$R^{I255d/+}$ - NAM 1000	2.67 ± 0.59	$p = 0.9974$	6				P
			$R^{I255d/+}$ - NAM 2000	2.53 ± 0.68	$p = 0.9637$	5				P
Fig. 6F	TUNEL	$R^{I255d/+}$ - NT	WT - NT	2.58 ± 0.36	$p = 0.0019$	6	Between (5) Residual (19)	F (5, 19) = 19.27	$p < 0.0001$	P
			$R^{I255d/+}$ - NT	6.48 ± 2.36		13				P
			$R^{I255d/+}$ - NAM 20	6.82 ± 0.88	$p = 0.8127$	6				P
			$R^{I255d/+}$ - NAM 200	9.10 ± 1.30	$p = 0.0431$	6				P
			$R^{I255d/+}$ - NAM 1000	12.77 ± 2.89	$p = 0.0002$	6				P
			$R^{I255d/+}$ - NAM 2000	12.97 ± 2.05	$p < 0.0001$	5				P

Table S9. Effect of DMSO and PARP inhibitors, including olaparib (Ola), saruparib (Sar), INO1001 (INO), and nicotinamide (NAM), on outer nuclear layer (ONL) rows counts, with different concentrations, DMSO (0.01, 0.1 and 1%), Ola, Sar and INO (0.1, 1 and 1μM), and NAM (20, 200, 1000 and 2000 μM): Quantitative data for graphs presented in Figures 7D. Test = Shapiro-Wilk normality test, P = pass, N = Not pass.

Table S9	*p* - value comparison	Genotype-treatment	Mean ± SD (%)	*p* - value	n	DF (column)	F – value	*p* - value	Test
Fig. 7D ONL rows	NT	WT – NT	8.46 ± 1.66		9	Between (3) Residual (16)	F (3, 16) = 1.516	*p* = 0.2487	P
		WT – DMSO 0.01	10.18 ± 0.44	*p* = 0.1565	4				P
		WT – DMSO 0.1	9.58 ± 1.74	*p* = 0.5510	3				P
		WT – DMSO 1	9.23 ± 1.06	*p* = 0.7289	4				P
	NT	$R^{I255d/+}$ - NT	4.83 ± 1.07		16	Between (3) Residual (21)	F (3, 21) = 2.084	*p* = 0.1329	P
		$R^{I255d/+}$ - DMSO 0.01	5.73 ± 0.47	*p* = 0.3541	3				P
		$R^{I255d/+}$ - DMSO 0.1	5.87 ± 0.64	*p* = 0.2467	3				P
		$R^{I255d/+}$ - DMSO 1	5.82 ± 0.17	*p* = 0.2795	3				P
	$R^{I255d/+}$ - NT	WT – NT	8.46 ± 1.66	*p* < 0.0001	9	Between (4) Residual (29)	F (4, 29) = 19.26	*p* < 0.0001	P
		$R^{I255d/+}$ - NT	4.92 ± 1.09		14				P
		$R^{I255d/+}$ - Ola 0.1	5.42 ± 0.82	*p* = 0.9301	9				P
		$R^{I255d/+}$ - Ola 1	5.53 ± 0.80	*p* = 0.8301	9				P
		$R^{I255d/+}$ - Ola 10	4.24 ± 1.39	*p* = 0.1423	6				P
	$R^{I255d/+}$ - NT	WT – NT	8.46 ± 1.66	*p* < 0.0001	9	Between (4) Residual (19)	F (4, 19) = 20.73	*p* < 0.0001	P
		$R^{I255d/+}$ - NT	4.93 ± 1.14		14				P
		$R^{I255d/+}$ - Sar 0.1	4.99 ± 0.94	*p* = 0.5097	5				P
		$R^{I255d/+}$ - Sar 1	4.48 ± 0.46	*p* = 0.1509	5				P
		$R^{I255d/+}$ - Sar 10	4.60 ± 0.38	*p* = 0.2215	4				P
	$R^{I255d/+}$ - NT	WT – NT	8.46 ± 1.66	*p* < 0.0001	9	Between (4) Residual (18)	F (4, 18) = 19.09	*p* < 0.0001	P
		$R^{I255d/+}$ - NT	4.93 ± 1.09		14				P
		$R^{I255d/+}$ - INO 0.1	4.22 ± 0.39	*p* = 0.1958	6				P
		$R^{I255d/+}$ - INO 1	4.37 ± 0.87	*p* = 0.2616	4				P
		$R^{I255d/+}$ - INO 10	4.44 ± 0.17	*p* = 0.3406	3				P
	$R^{I255d/+}$ - NT	WT – NT	8.46 ± 1.66	*p* < 0.0001	9	Between (5) Residual (27)	F (5, 27) = 12.03	*p* < 0.0001	P
		$R^{I255d/+}$ - NT	4.96 ± 1.10		14				P
		$R^{I255d/+}$ - NAM 20	5.40 ± 0.70	*p* = 0.5134	6				P
		$R^{I255d/+}$ - NAM 200	5.39 ± 0.86	*p* = 0.5273	6				P
		$R^{I255d/+}$ - NAM 1000	5.62 ± 0.55	*p* = 0.2740	6				P
		$R^{I255d/+}$ - NAM 2000	5.56 ± 0.59	*p* = 0.4456	5				P
